# Persistence versus Escape: *Aspergillus terreus* and *Aspergillus fumigatus* Employ Different Strategies during Interactions with Macrophages

**DOI:** 10.1371/journal.pone.0031223

**Published:** 2012-02-03

**Authors:** Silvia Slesiona, Markus Gressler, Michael Mihlan, Christoph Zaehle, Martin Schaller, Dagmar Barz, Bernhard Hube, Ilse D. Jacobsen, Matthias Brock

**Affiliations:** 1 Microbial Biochemistry and Physiology, Leibniz Institute for Natural Product Research and Infection Biology, Hans Knoell Institute, Jena, Germany; 2 Microbial Pathogenicity Mechanisms, Leibniz Institute for Natural Product Research and Infection Biology, Hans Knoell Institute, Jena, Germany; 3 Infection Biology, Leibniz Institute for Natural Product Research and Infection Biology, Hans Knoell Institute, Jena, Germany; 4 Biomolecular Chemistry, Leibniz Institute for Natural Product Research and Infection Biology, Hans Knoell Institute, Jena, Germany; 5 Friedrich Schiller University, Jena, Germany; 6 Department of Dermatology, Eberhard-Karls-University, Tübingen, Germany; 7 Institute for Transfusion Medicine, University Hospital, Jena, Germany; Universidade de Sao Paulo, Brazil

## Abstract

Invasive bronchopulmonary aspergillosis (IBPA) is a life-threatening disease in immunocompromised patients. Although *Aspergillus terreus* is frequently found in the environment, *A. fumigatus* is by far the main cause of IBPA. However, once *A. terreus* establishes infection in the host, disease is as fatal as *A. fumigatus* infections. Thus, we hypothesized that the initial steps of disease establishment might be fundamentally different between these two species. Since alveolar macrophages represent one of the first phagocytes facing inhaled conidia, we compared the interaction of *A. terreus* and *A. fumigatus* conidia with alveolar macrophages. *A. terreus* conidia were phagocytosed more rapidly than *A. fumigatus* conidia, possibly due to higher exposure of β-1,3-glucan and galactomannan on the surface. In agreement, blocking of dectin-1 and mannose receptors significantly reduced phagocytosis of *A. terreus*, but had only a moderate effect on phagocytosis of *A. fumigatus*. Once phagocytosed, and in contrast to *A. fumigatus*, *A. terreus* did not inhibit acidification of phagolysosomes, but remained viable without signs of germination both *in vitro* and in immunocompetent mice. The inability of *A. terreus* to germinate and pierce macrophages resulted in significantly lower cytotoxicity compared to *A. fumigatus*. Blocking phagolysosome acidification by the v-ATPase inhibitor bafilomycin increased *A. terreus* germination rates and cytotoxicity. Recombinant expression of the *A. nidulans wA* naphthopyrone synthase, a homologue of *A. fumigatus* PksP, inhibited phagolysosome acidification and resulted in increased germination, macrophage damage and virulence in corticosteroid-treated mice. In summary, we show that *A. terreus* and *A. fumigatus* have evolved significantly different strategies to survive the attack of host immune cells. While *A. fumigatus* prevents phagocytosis and phagolysosome acidification and escapes from macrophages by germination, *A. terreus* is rapidly phagocytosed, but conidia show long-term persistence in macrophages even in immunocompetent hosts.

## Introduction

Invasive bronchopulmonary aspergillosis (IBPA) is a life-threatening disease mainly caused by *Aspergillus fumigatus* [1]. Additionally, several other *Aspergillus* species, including *A. terreus*, have been identified as causative agents [2]. Approximately 3–12.5% of IBPA cases are caused by *A. terreus* [3], [4], [5] with dramatic mortality rates of up to 98% [6], [7]. Therapy of IBPA caused by *A. terreus* is complicated by its intrinsic resistance to Amphotericin B [8], [9] and prophylactic Amphotericin B monotherapy in high risk patients results in an increased likelihood to acquire IBPA caused by *A. terreus* [10]. In addition, invasive aspergillosis due to *A. terreus* is accompanied by a high rate of dissemination to secondary organs further complicating treatment of affected patients [5], [11].

*Aspergillus* conidia are ubiquitous and, due to their small size, reach the lower airways upon inhalation. In the immunocompetent host, these spores are cleared by phagocytic immune cells [12]. If the phagocytic immune defense is impaired, e.g. by prolonged corticosteroid therapy or chemotherapy, conidia are able to germinate and form hyphae which invade the lung tissue and establish an infection [13], [14].

Alveolar macrophages (AM) are the first professional phagocytic cells encountering pathogens in the lung. In addition to eliminating pathogens by phagocytosis, AM participate in orchestrating the immune response [15]. Therefore, the initial step of successful infection depends on the pathogen's ability to prevent or survive recognition and phagocytosis by macrophages. Consequently, pathogens have developed diverse escape and survival strategies [16], [17], [18], [19]. The interaction of *A. fumigatus* with macrophages has been well studied: In immunocompetent mice, AM phagocytose *A. fumigatus* conidia and control fungal burden unless overwhelmed by a large number of conidia [20], [21], demonstrating the relevance of *A. fumigatus* – macrophage interaction for the clearance of inhaled *A. fumigatus* conidia. The pathogen recognition receptors (PRRs) dectin-1, TLR-2 and TLR-4 [15], [21], [22], [23] are involved in this interaction, recognizing β-1,3-glucan and yet unidentified pathogen-associated molecular patterns (PAMPs), respectively. Survival of *A. fumigatus* upon phagocytosis depends on its ability to inhibit phagolysosome acidification, which allows a rapid germination and escape from the phagocyte. Blocking of phagolysosome acidification depends on the presence of DHN-melanin on the surface of *A. fumigatus* conidia [24], [25].

Although *A. terreus* is frequently found in the environment [26], IBPA caused by this fungus is less common than infections caused by *A. fumigatus*. However, the outcome of *A. terreus* infections is even more often fatal than *A. fumigatus* infections [5], [27]. Therefore, we investigated the initial steps of disease establishment, hypothesizing that these might be fundamentally different between the two species. Since alveolar macrophages represent one of the first phagocytes facing inhaled conidia, we investigated this interaction of *A. fumigatus* and *A. terreus* by comparing phagocytosis, inactivation of conidia and phagolysosome maturation. Using living conidia and time course experiments, we demonstrate significant differences between both species on several levels: phagocytosis of conidia, phagolysosome maturation, fungal viability after phagocytosis, escape from macrophages and production of pro-inflammatory cytokines. Furthermore, we investigated the accessibility of conidial PAMPs and the impact of a melanin precursor for intracellular growth. We propose that the observed differences between both fungi might attribute to long-term survival of *A. terreus* conidia within macrophages leading to outbreak of invasive aspergillosis after profound immunosuppression.

## Results

### *A. terreus* is phagocytosed more rapidly than *A. fumigatus*

Upon inhalation, alveolar macrophages are the first professional phagocytic cells facing fungal conidia in the lower respiratory tract. We proposed that the different efficiency of phagocytosis and intracellular killing of *A. fumigatus* or *A. terreus* conidia by macrophages might contribute to the different epidemiology of these two fungi. While phagocytosis of *A. fumigatus* conidia by macrophages has been extensively studied [28], [29], [30], little is known about the phagocytosis of *A. terreus* conidia. Therefore, we compared phagocytosis of both *Aspergillus* species using alveolar and peritoneal macrophage cell lines (MH-S and J774.A1, respectively).

**Figure 1:**
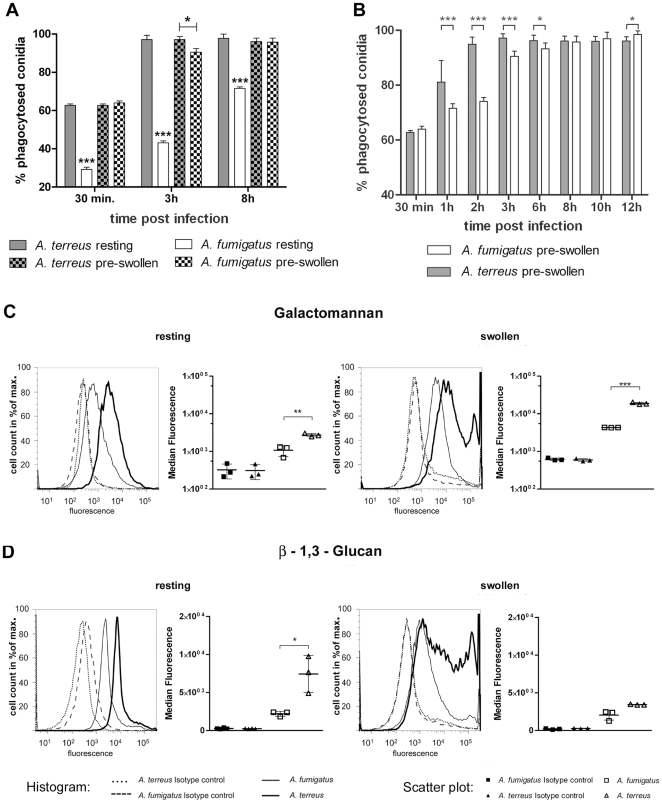
Phagocytosis rates and surface exposure of galactomannan and β-1,3-glucan on resting and swollen *A. terreus* and *A. fumigatus* conidia. Results are shown for *A. terreus* SBUG844 and *A. fumigatus* CBS144.89. (A, B) Phagocytosis of conidia by alveolar macrophages (MH-S cell line). Phagocytosis rates are shown as mean+SD from three independent experiments. Statistical analysis was performed using 1-way ANOVA. * P<0.05, ** P<0.01, *** P<0.001. (A) Phagocytosis of resting and pre-swollen conidia after 0.5, 3 and 8 h of co-incubation. (B) Phagocytosis of pre-swollen conidia over 12 h. (C) FACS analysis of galactomannan and (D) β-1,3-glucan exposure. Histograms (one representative result from three independent experiments is shown) and scatter plots of median fluorescence intensity are shown (mean ± SD, three independent experiments). Statistical analysis was performed using paired t-test. * P<0.05; ** P<0.01; *** P<0.001. Dashed and dotted lines in the histogram show the respective isotype controls while the full lines represent specific fluorescence for *A. fumigatus* (thin) and *A. terreus* (thick).

Resting *A. terreus* conidia were phagocytosed significantly faster than resting *A. fumigatus* conidia by both MH-S (Fig. 1A) and J774.A1 cells (data not shown). While phagocytosis of resting *A. terreus* conidia was completed after three hours (96% phagocytosed), the ratio of phagocytosed resting *A. fumigatus* conidia continued to increase for at least eight hours (Fig. 1A).

Since resting *Aspergillus* conidia swell in the cell culture media, we additionally determined the phagocytosis of pre-swollen conidia. Preliminary experiments revealed that *A. terreus* conidia require a prolonged time for germination and possess a lower growth speed than *A. fumigatus*. To account for these differences, the swelling and germination speed of both fungi was evaluated in different cell culture media (Fig. S1). Swelling was judged by microscopic and flow cytometry analysis using the (i) increase of conidial diameter, (ii) opacity of conidia and (iii) the loss of spore coloration as markers. Homogenous populations containing >95% swollen conidia but no germlings were obtained for *A. fumigatus* by 3.5 h pre-incubation in RPMI and 6 h pre-incubation in DMEM medium, respectively. Due to slower swelling of *A. terreus* conidia, pre-incubation for 7 h in RPMI and 10 h in DMEM medium were required to obtain homogenous populations of swollen conidia (Fig. S1). Since phagocytosis can be influenced by particle size, we additionally determined the size of resting and pre-swollen conidia using FACS. The diameter of both resting and swollen *A. terreus* conidia was smaller than that of *A. fumigatus* conidia (Fig. S2).

Pre-swelling led to a significant increase in the phagocytosis of *A. fumigatus* conidia. In contrast, phagocytosis of *A. terreus* conidia was not affected by pre-swelling (Fig. 1A). Using pre-swollen conidia, no difference between *A. terreus* and *A. fumigatus* was observed for initial (30 min) and late phagocytosis (8 h). However, we observed significant differences between 1 h to 3 h. Therefore, we analyzed phagocytosis of pre-swollen conidia on a more detailed time scale (Fig. 1B). Significant differences in the proportion of phagocytosed pre-swollen conidia were observed between 1 h and 6 h.

### β-1,3-glucan and galactomannan significantly contribute to pathogen recognition in the phagocytosis of *A. terreus*

It has been shown that phagocytosis of *A. fumigatus* conidia depends on the exposure of pathogen associated molecular patterns (PAMPs) such as β-1,3-glucan and galactomannan [31]. PAMPs are recognized by pathogen recognition receptors (PRRs) located on the surface of macrophages. Known PRRs involved in recognition of fungi include dectin-1 recognizing β-1,3-glucan [32], mannose receptors recognizing galactomannan [33], [34], and the toll like receptors TLR2 and TLR4 recognizing yet unknown factors [35]. We hypothesized that the observed differences in phagocytosis rates could result from differences in PAMP exposure and PRR recognition.

Therefore, we quantified β-1,3-glucan and galactomannan exposure on the surface of resting and swollen conidia by flow cytometry using specific antibodies (kindly provided by A. Cassone and F. Ebel). Resting conidia of *A. terreus* displayed significantly higher exposure of β-1,3-glucan (as recently shown by Deak et al. [36]) and galactomannan than resting *A. fumigatus* conidia. As expected, swelling of conidia increased the exposure of galactomannan in both fungi as indicated by an increase in specific fluorescence (Fig. 1C). The pre-swelling conditions applied to both fungi did not significantly increase the exposure of β-1,3-glucan determined by mean fluorescence levels (Fig. 1D). This confirmed that no germ tubes were formed, which have been shown to display high β-1,3-glucan levels [37]. Importantly, swollen conidia of *A. terreus* showed higher exposure of both β-1,3-glucan and galactomannan than *A. fumigatus*. However, within the *A. terreus* population, β-1,3-glucan exposure was distributed inhomogenously.

**Figure 2:**
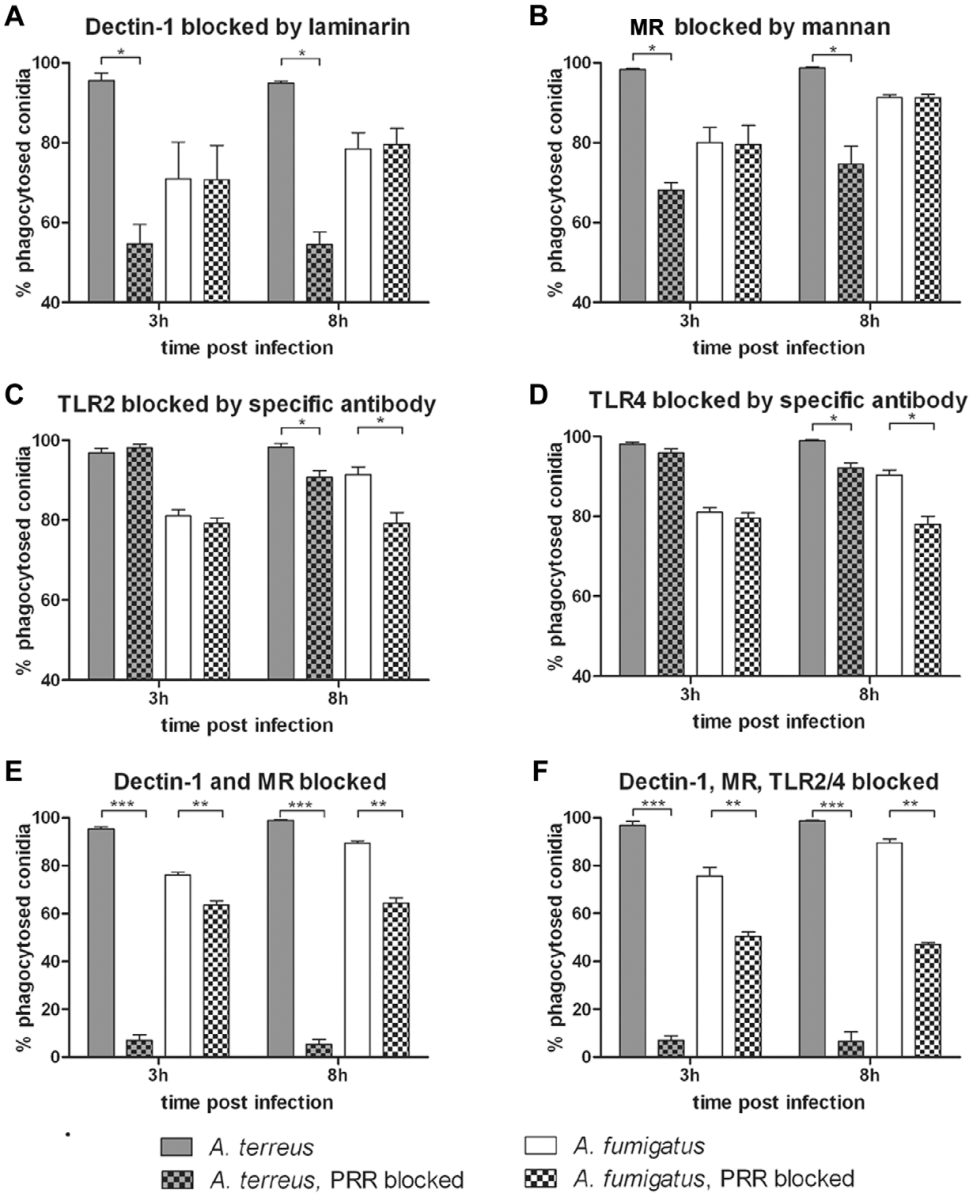
Role of PRRs in phagocytosis of pre-swollen *A. terreus* and *A. fumigatus* conidia by MH-S cells. Results are shown for *A. terreus* SBUG844 and *A. fumigatus* CBS144.89. Phagocytosis rates are shown as mean+SD from three independent experiments. Statistical analysis was performed using 1-way ANOVA. * P<0.05, ** P<0.01, *** P<0.001. (A) Blocking of dectin-1 by laminarin. (B) Blocking of mannose receptors (MR) by mannan. (C) Blocking of TLR2 or (D) of TLR4 by specific antibodies. (E) Simultaneous blocking of dectin-1 and mannose receptors. (F) Simultaneous blocking of dectin-1, mannose receptors, TLR2 and TLR4.

To investigate the role of different PAMPs, we determined phagocytosis of pre-swollen conidia 3 h and 8 h after blocking the receptors of MH-S cells by specific substrates or specific antibodies. Pre-swollen conidia were chosen for these and subsequent *in vitro* experiments because differences in phagocytosis rates between *A. fumigatus* and *A. terreus* were less pronounced for pre-swollen compared to resting conidia. Blocking of dectin-1 by laminarin and blocking of mannose receptors by mannan significantly decreased the phagocytosis of *A. terreus* conidia within the first three hours by 50% and 30%, respectively. The overall phagocytosis rate of *A. terreus* did not further increase after prolonged co-incubation (Fig. 2A and 2B). In contrast, blocking of either dectin-1 or mannose receptors did not influence phagocytosis of *A. fumigatus* (Fig. 2A and 2B). These data suggested that recognition by dectin-1 and mannose receptors played a major role in the early recognition of *A. terreus*, but not *A. fumigatus*. Blocking of either TLR2 or TLR4 led to a moderate but significant reduction in phagocytosis, but these differences were only observed after 8 h (Fig. 2C and 2D). The influence of TLR2 and TLR4 on the long-term phagocytosis of pre-swollen *A. fumigatus* conidia coincided with the occurrence of germlings (Fig. S1), suggesting that the ligands for TLR2/4 are either exposed on germlings or that the recruitment of PRRs to the phagosome and the subsequent antigen presentation affects long-term phagocytosis [15]. It appeared possible that blocking of a single receptor can be bypassed by other receptors. Thus, we performed simultaneous blocking of mannose receptors and dectin-1 as well as simultaneous blocking of all four tested receptors. Simultaneous blocking of mannose receptors and dectin-1 nearly completely abolished phagocytosis of *A. terreus*, but had only a moderate effect on *A. fumigatus* (>60% phagocytosed, Fig. 2E). Blocking of all receptors did not further reduce phagocytosis of *A. terreus* (Fig. 2F), but further decreased phagocytosis of *A. fumigatus* conidia (50% phagocytosed, Fig. 2F). These data confirm that, at least in MH-S cells, dectin-1 and mannose receptors are the main PRRs recognizing *A. terreus* conidia, whereas different and probably yet unknown receptors are involved in phagocytosis of *A. fumigatus* conidia.

### Viable *A. terreus* conidia persist in macrophages after phagocytosis

Phagocytosis is one of the first steps in the inactivation of many microbial pathogens. However, some microbes are able to escape killing after phagocytosis [37], [38], [39]. Thus, we investigated which proportion of phagocytosed pre-swollen conidia were inactivated.

**Figure 3:**
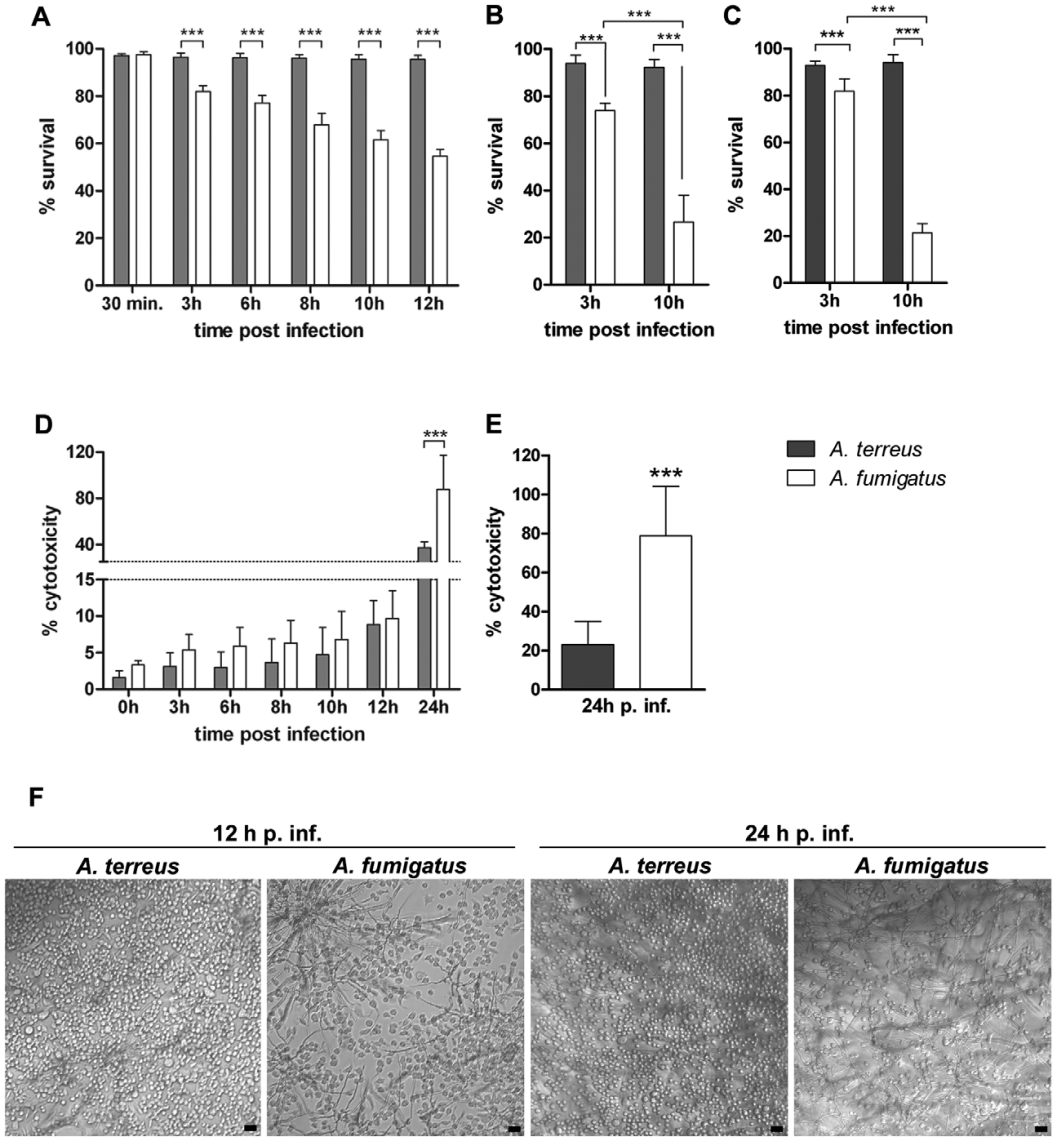
*A. fumigatus* and *A. terreus* survival, host cell damage and hyphal growth in co-incubation with macrophages. Results are shown for *A. terreus* SBUG844 and *A. fumigatus* CBS144.89. (A–C) Survival of *A. fumigatus* and *A. terreus* upon co-incubation with macrophages. Data is shown as mean+SD from three independent experiments; statistical analysis by 1-way ANOVA and Tukey's multiple comparison test. * P<0.05; ** P<0.01; *** P<0.001. (A, B) Co-incubation with MH-S cells and (C) with bone marrow-derived murine macrophages (A) Survival of *A. terreus* and *A. fumigatus* determined by fluorescence microscopy using the metabolic dye FUN1. (B, C) Survival determined by CFU. (D, E) Relative cytotoxicity of *A. terreus* and *A. fumigatus* conidia to (D) MH-S cells and (E) human monocyte-derived macrophages determined by release of lactate dehydrogenase (LDH). Mean values+SD from three independent experiments; statistical analysis was performed per time point by unpaired, two-tailed t-test. * P<0.05; ** P<0.01; *** P<0.001 (F) Microscopic picture showing germination and filamentous growth of *A. fumigatus* and *A. terreus* 12 h (left) and 24 h (right) after infection. Scale bars = 40 µm.

In a first set of experiments, we discriminated dead and viable conidia on a single-conidia level by using a fluorescent dye, which indicates metabolic activity of cells. The ratio of inactivated *A. fumigatus* conidia steadily increased over time after phagocytosis by MH-S cells (Fig. 3A). Furthermore, in addition to the metabolic inactivation indicated by color changes of the dye, *A. fumigatus* conidia appeared deformed, suggesting that conidia were not only inactivated, but also partially degraded. In striking contrast, no inactivation of *A. terreus* conidia was observed (Fig. 3A), indicating an increased resistance of *A. terreus* within macrophages. Similar results were obtained using J774.A1 cells (data not shown). In addition, we determined survival by counting colony forming units (CFU) 3 h and 10 h after infection. *A. fumigatus* showed the expected time dependent inactivation, while *A. terreus* CFUs remained stable (Fig. 3B). Increased survival of *A. terreus* was likewise observed upon interaction with primary mouse macrophages (Fig. 3C). To confirm that differences in survival rates were not strain but species specific, two additional strains from each species were investigated for their survival within MH-S cells. No significant differences between species specific strains were observed, confirming that our findings are not limited to a specific strain (Fig. S3).

*A. fumigatus* conidia which are not inactivated after phagocytosis tend to form germ tubes that pierce macrophages and eventually lead to lysis of phagocytic cells [40]. To investigate whether the increased persistence of *A. terreus* conidia in macrophages was likewise associated with host cell damage, we determined the LDH release from macrophages as a marker for macrophage damage. We used pre-swollen conidia to minimize effects caused by the overall slower germination (Fig. S1) and growth speed of *A. terreus* compared to *A. fumigatus*, which could affect damage of macrophages by *A. terreus* in co-culture experiments [41]. Furthermore, we analyzed fungal germination and growth in cell culture medium in the absence of macrophages. No gross differences between *A. fumigatus* and *A. terreus* were observed in these experiments (Fig. S4A). In co-incubation with macrophages, only mild cell damage was observed with either of the two fungi within the first 12 h. However, cell damage caused by *A. fumigatus* strongly increased 24 h post infection, whereas *A. terreus* caused significantly less damage. This difference was significant after 24 h in MH-S cells (Fig. 3D), independent of the *A. terreus* and *A. fumigatus* strain used (Fig. S4B). The difference in cytotoxicity caused by *A. terreus* and *A. fumigatus*, respectively, was even more pronounced in human primary macrophages (Fig. 3E).

Microscopy revealed that phagocytosed *A. fumigatus* conidia developed hyphae, which pierced macrophages within the first 12 h (Fig. 3F). In contrast, germination of phagocytosed *A. terreus* conidia was not observed after 12 h and rarely after 24 h (Fig. 3F). Non-phagocytosed conidia (approximately 5% of both *A. fumigatus* and *A. terreus* total conidia) of both species formed filaments to the same extent (Fig. S4A). Thus, we propose that, despite the high survival rate of *A. terreus* conidia, the slow germination of phagocytosed *A. terreus* conidia compared to *A. fumigatus* led to low macrophage damage.

### Phagolysosomes containing *A. terreus* conidia mature and acidify

Upon phagocytosis, phagosomes mature *via* a series of fusion events to mature, acidified phagolysosomes. The fusion events are characterized by the recruitment of several marker proteins, including LAMP-1 (lysosome-associated membrane protein) and Cathepsin D (lysosomal aspartyl protease) [42]. In fully matured phagolysosomes, v-ATPase within the membrane builds up a proton gradient resulting in a pH decrease. The low pH provides an optimal environment for phagolysosomal enzymes and is essential for successful killing of most pathogens [43] including *A. fumigatus* [24], [25]. Viable *A. fumigatus* conidia inhibit this acidification [24], [25], allowing the fungus to germinate within the phagolysosome and to escape by piercing through the macrophage membrane as shown previously [44] and in our experiments described above (Fig. 3). To determine whether the observed differences in fungal inactivation of *A. terreus* and *A. fumigatus* by macrophages and the observed macrophage damage might be related to differences in phagosome maturation, we analyzed phagosome maturation using fluorescence microscopy.

**Figure 4:**
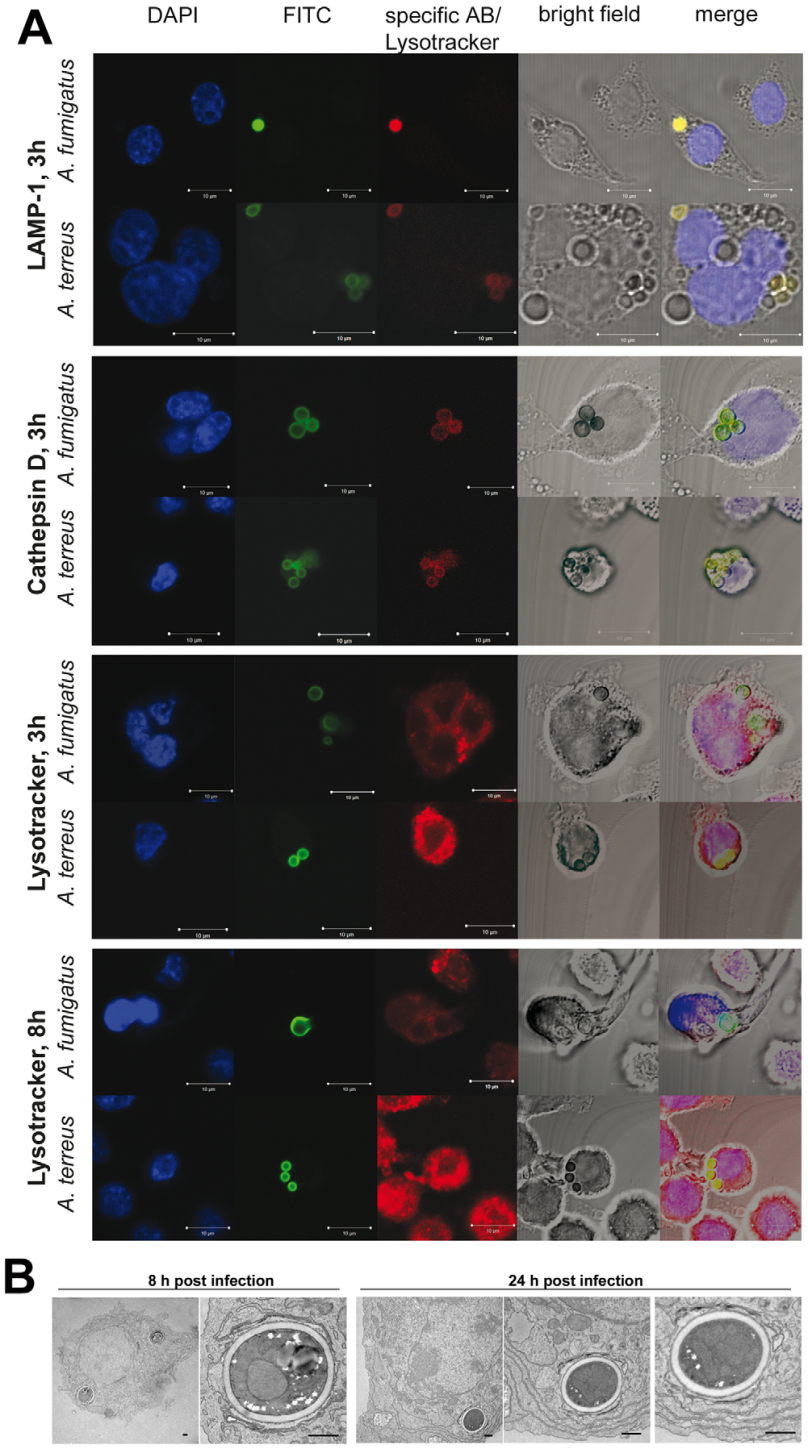
Maturation of phagolysosomes containing *A. fumigatus* or *A. terreus* conidia. Results are shown for *A. terreus* SBUG844 and *A. fumigatus* CBS144.89 with MH-S cells. (A) FITC labeled conidia were used. Yellow signal in the merged pictures indicate co-localization of conidia with the specific phagolysosome stain. All lanes show representative fluorescence microscopy pictures. Blue: DAPI (nucleus); green: FITC labeled conidia; red: specific antibody for the phagolysosomal marker indicated on the left. Bars represent a size of 10 µm. (B) Transmission electron micrograph of phagocytosed *A. terreus* conidia 8 h and 24 h after infection. Bars represent 0.5 µm. Phagocytosed conidia are surrounded by a phospholipid bilayer at both time points.

Phagosomes of MH-S cells containing pre-swollen conidia of either *A. fumigatus* or *A. terreus* successfully underwent fusion with lysosomes 3 h (Fig. 4A) and 8 h (data not shown) after infection as indicated by the presence of LAMP-1 and Cathepsin D, characterizing a mature phagolysosome [45]. However, by using the acidic probe Lysotracker DND99 we observed that only phagolysosomes containing *A. terreus* conidia were invariably (>99%) acidified at both time points, whereas few *A. fumigatus* conidia were found in acidified phagolysosomes (10–15% after 3 h; 25–30% after 8 h) (Fig. 4A). All *A. fumigatus* conidia in acidified phagosomes looked deformed. Similar results were obtained using primary murine alveolar macrophages and primary human monocyte-derived macrophages (Fig. S5). Inhibition of acidification by *A. fumigatus* and acidification of phagolysosomes containing *A. terreus* was likewise observed with two additional strains of each *A. fumigatus* and *A. terreus* (Fig. S6). Thus, in combination with our observation that phagocytosed *A. terreus* conidia remain viable, these findings suggest that *A. terreus* does not block acidification but survives in acidified phagolysosomes. The persistence of intact *A. terreus* conidia within phagolysosomes was additionally confirmed by transmission electron microscopy 8 h and 24 h after infection (Fig. 4B).

### *A. terreus* is trapped in the non-germinated stage but resistant to acidic pH

**Figure 5:**
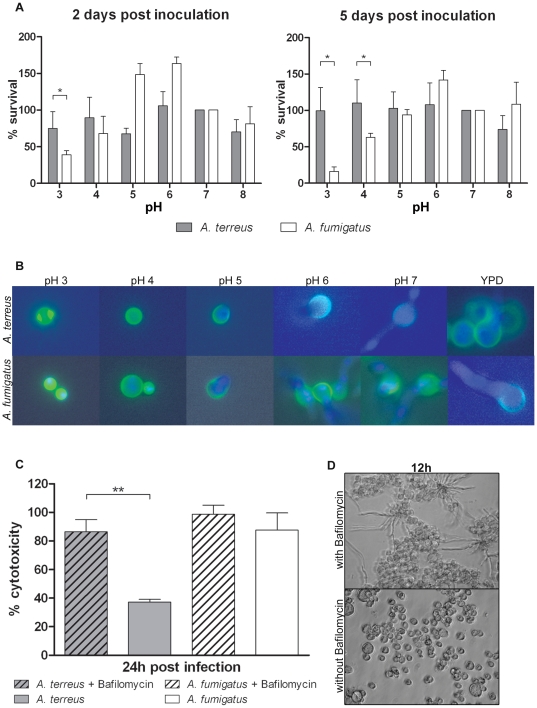
Influence of environmental pH on *in vitro* survival, germination and effect of bafilomycin on macrophage lysis. Results are shown for *A. terreus* SBUG844 and *A. fumigatus* CBS144.89. Data shown represent mean+SD from three independent experiments. Statistical analysis was performed using paired t-test (A, C; * P<0.05) or display representative pictures (B, D) from three independent experiments. (A) Relative survival of *A. terreus* and *A. fumigatus* conidia after incubation in nutrient-free, pH-stabilized buffer. Survival at pH 7 is set to 100%. Left panel: survival after two days, right panel: survival after five days. (B) Germination of *A. terreus* and *A. fumigatus* conidia in minimal medium with defined pH. All pictures were acquired after 8 h incubation at 37°C. (C) Relative cytotoxicity (LDH release) mediated by *A. terreus* and *A. fumigatus* conidia on alveolar macrophages treated with the v-ATPase blocker bafilomycin or without bafilomycin treatment. (D) Brightfield microscopy of *A. terreus* in co-incubation with macrophages (MH-S) without (upper lane) and with bafilomycin (lower lane) 12 h. Bafilomycin treatment allows *A. terreus* to germinate and escape from macrophages.

Since *A. terreus* conidia were not inactivated after phagocytosis, we hypothesized that resistance to acidic pH may enable *A. terreus* to survive in the phagolysosome. Thus, we determined the *in vitro* survival of conidia from both species in phosphate buffer at different pH values without addition of nutrients, a condition which may closely resemble that in phagolysosomes. In this nutrient-limited environment, no germination of either *Aspergillus* species was observed. However, *A. terreus* survived incubation at pH 3 and 4 to a higher extent than *A. fumigatus* (Fig. 5A). The addition of nutrients showed that both species germinated at pH values>5, whereas swelling and germination of conidia was suppressed at lower pH (Fig. 5B). Thus, we assume that while *A. terreus* conidia survived within the acidified phagolysosome, they were unable to germinate, did not pierce the macrophage membranes and caused less cell damage. In contrast, the ability of *A. fumigatus* to inhibit phagolysosome acidification allowed rapid germination of conidia at ambient pH, which caused the high cytotoxicity observed after 24 h (Fig. 3).

To determine the effect of phagolysosome acidification on conidial germination, we investigated macrophage damage in the presence of bafilomycin. Bafilomycin is a potent v-ATPase blocker, which inhibits the acidification of phagolysosomes [46]. Treatment with bafilomycin significantly increased cell damage caused by *A. terreus* (Fig. 5C) to the level observed in macrophages incubated with *A. fumigatus* conidia in the absence of bafilomycin. In contrast, only a slight increase in cell damage by *A. fumigatus* was observed after bafilomycin treatment (Fig. 5C). In agreement with increased macrophage damage, *A. terreus* showed faster germination and piercing of macrophages in bafilomycin-treated macrophages (Fig. 5D). These differences were also observed in other *A. fumigatus* and *A. terreus* strains tested (Fig. S7A). In the absence of macrophages, bafilomycin had no influence on fungal germination and growth (Fig. S7B and data not shown). These results support the hypothesis that inhibition of germination is due to acidification of phagolysosomes, which prevents escape and the accompanied macrophage damage by *A. terreus*.

### Phagocytosed *A. terreus* conidia survive in the lungs of immunocompetent mice

**Figure 6:**
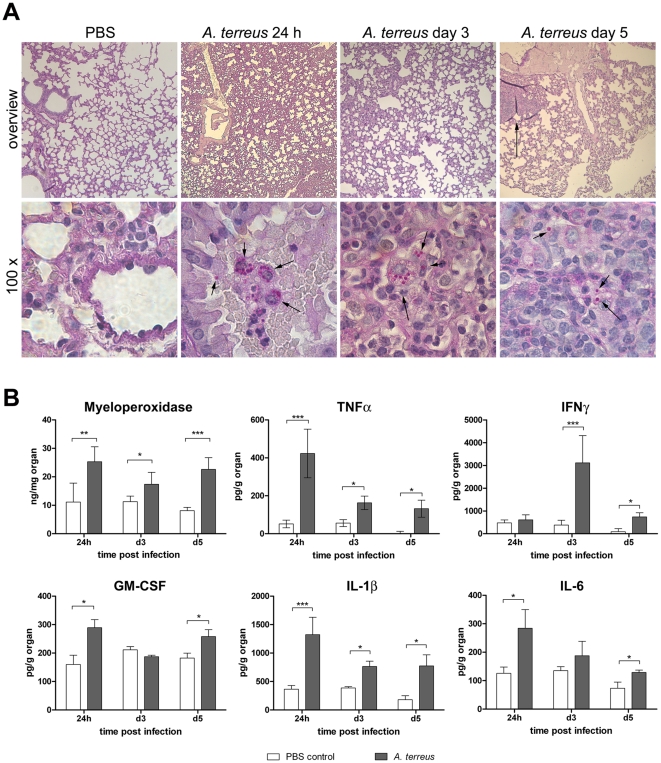
Persistence of *A. terreus* conidia in the lungs of immunocompetent mice. Infection was performed with *A. terreus* SBUG844. (A) Histological sections, Periodic-Acid-Schiff stain. 24 h, day 3, day 5: time after infection. Upper row: overview 4× magnified; the arrow indicates immune cell infiltrate. Lower row: 100× magnified; arrows indicate *A. terreus* conidia stained in pink. Scale bars represent 100 µm (overview) and 10 µm (100×). (B) Immune response in the lungs determined by myeloperoxidase and cytokine levels. White bars: PBS-mock infected mice; grey bars: *A. terreus* infected mice. Data shown as mean+SD from 5 animals per group. Statistical analysis was performed per time point by unpaired, two-tailed t-test. * P<0.05; ** P<0.01; *** P<0.001.

Our *in vitro* data suggested that *A. terreus* conidia are able to survive for a prolonged time within macrophages. To determine whether persistence in macrophages also occurs *in vivo*, we infected immunocompetent mice intranasally with 1×10^7^ *A. terreus* conidia. All animals remained clinically healthy and displayed no macroscopic alterations at necropsy 24 h, 3 days and 5 days after infection. Semiquantitative isolation of *A. terreus* from the lungs of these animals revealed persistently high fungal burdens of 5×10^5^ CFU and higher without differences between the time points. Histologically, un-swollen but intact conidia within macrophages could be observed in the lungs at all time points (Fig. 6A). No germlings or hyphae were found. The lung tissue itself appeared unaltered 24 h and 3 days after infection; however, in all infected mice analyzed on day 5, small, single foci of mononuclear cell infiltrates were found in the lungs (Fig. 6A). The levels of myeloperoxidase (as neutrophil marker), TNFα, INFγ, GM-CSF, IL-1β and IL-6 transiently increased in lungs after infection (Fig. 6B), while levels of IL-10, IL-17A and IL-22 were indistinguishable from PBS controls. Thus, *A. terreus* conidia persist *in vivo* for several days within macrophages in the lung despite a detectable immune response.

### Recombinant expression of the *A. nidulans wA* gene allows *A. terreus* to prevent phagolysosome acidification

The ability of *A. fumigatus* to inhibit phagolysosome acidification is linked to the grey-greenish spore coloration derived from DHN-melanin [25], [47]. Deletion of the polyketide synthase gene *pksP* in *A. fumigatus* inhibits melanin synthesis and the *pksP* mutant is not able to block phagolysosome acidification, resulting in increased susceptibility to killing by macrophages [25]. A homologous polyketide synthase responsible for conidia coloration is also present in other aspergilli, like *A. nidulans* or *Aspergillus niger* [48], [49]. In *A. nidulans*, the green spore color derives from the yellow melanin precursor naphthopyrone and is produced by the *wA* gene product [50], [51]. Interestingly, although PksP produces DHN-melanin in *A. fumigatus*, the product formed after recombinant expression of *pksP* in *Aspergillus oryza*e is naphthopyrone, which indicates that the initial products formed by *A. nidulans wA* and *A. fumigatus pksP* are identical [52].

Despite the brownish coloration of *A. terreus* conidia and several polyketide synthase genes present in its genome, *A. terreus* seems to lack a homologue of *pksP* and *wA* (Fig. S8). Consequently, *A. terreus* produces neither DHN-melanin nor naphthopyrone (Fig. S9C). While DHN-melanin seems essential for the inhibition of phagolysosome acidification by *A. fumigatus*, it has not yet been investigated whether naphthopyrone is also able to prevent acidification. Therefore, we tested the hypothesis that the lack of a *pksP*-homologue is responsible for the inability of *A. terreus* to inhibit phagolysosome acidification by introducing the *wA* gene from *A. nidulans* into the genome of *A. terreus*. The mutant with a single integration of the *wA* gene, denoted as *A. terreus wA*, showed a yellowish phenotype of conidia comparable to the color of an *A. nidulans yA* mutants, which lacks a *p*-diphenol oxidase polymerizing naphthopyrone [53] (Fig. S9A and S9B). HPLC-MS analysis confirmed that *A. terreus wA* produced the naphthopyrone derivatives YWA1 and YWA2 [50] in conidia (Fig. S9D).

**Figure 7:**
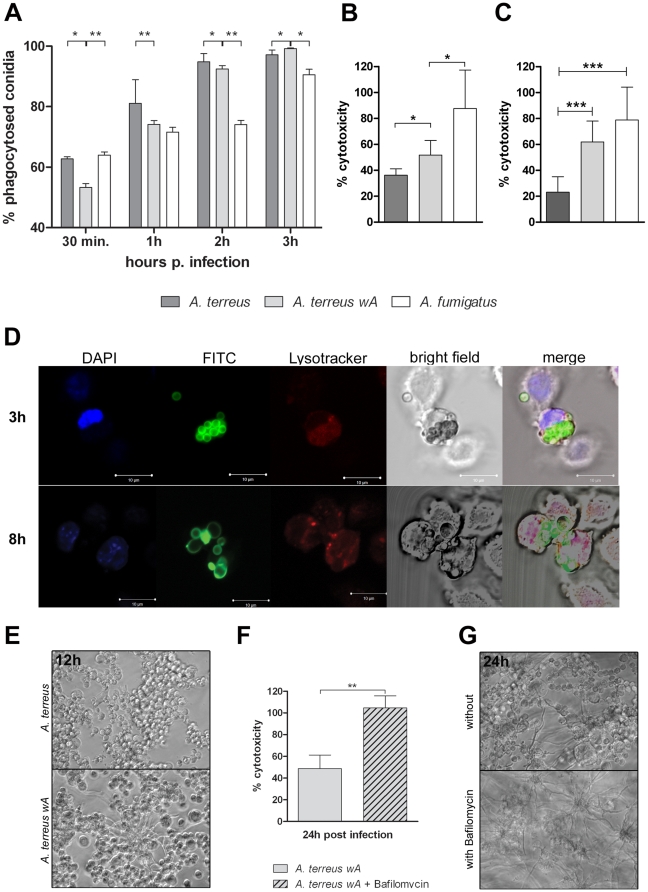
Interaction of the naphthopyrone producing strain *A. terreus wA* with macrophages. Unless stated otherwise, the alveolar macrophage cell line MH-S was used. (A) Phagocytosis of pre-swollen *A. terreus wA* compared to *A. terreus* wild type and *A. fumigatus* (mean+standard deviation of three independent experiments analyzed by 1-way ANOVA and Tukey's multiple comparison test; * P<0.05; ** P<0.01). During the first hour phagocytosis of *A. terreus wA* is delayed, but reaches the level of the wild type after 2 h of co-incubation. (B, C) Relative cytotoxicity (LDH release) mediated by *A. terreus wA* compared to *A. terreus* wild type and *A. fumigatus* (mean+standard deviation of three independent experiments analyzed by 1-way ANOVA and Tukey's multiple comparison test; * P<0.05). (B) MH-S cells. (C) Human monocyte-derived macrophages. (D) Acidification of phagolysosomes containing *A. terreus wA* conidia. Representative fluorescence microscopy pictures are shown. Blue: DAPI (nucleus); green: FITC labeled conidia; red: Lysotracker DND99. The white bars represent 10 µm. The staining revealed no phagolysosome specific signal in the red channel (conidia are not visible) 3 h and 8 h after infection. (E) Brightfield microscopy of *A. terreus* and *A. terreus wA* 12 h after infection. *A. terreus wA* escapes much faster from macrophages than the wild type and starts to form a mycelium layer. (F) Effect of bafilomycin compared to untreated macrophages on relative cytotoxicity (LDH release) mediated by *A. terreus wA* (mean+standard deviation from three independent experiments, unpaired, two-tailed t-test; ** P<0.01). (G) Brightfield microscopy of *A. terreus wA* 24 h after infection of untreated and bafilomycin-treated macrophages. With bafilomycin nearly all macrophages have been lysed by *A. terreus wA*.

Although initial phagocytosis of *A. terreus wA* conidia was slightly delayed in comparison to *A. terreus* wild type, phagocytosis rates after 2 h largely resembled the wild type (Fig. 7A). Thus, expression of *wA* in *A. terreus* did not seem to have a major impact on phagocytosis. Compared to the wild-type strain, *A. terreus wA* caused increased damage to MH-S cells, although damage was still significantly less than that caused by *A. fumigatus* (Fig. 7B). This increase in cytotoxicity was even more pronounced in human monocyte-derived macrophages (Fig. 7C). Consistent with the inhibitory role on phagolysosome acidification proposed for PksP in *A. fumigatus*, phagolysosomes containing viable *A. terreus wA* conidia did not acidify in MH-S cells (Fig. 7D), primary murine alveolar macrophages and human monocyte-derived macrophages (Fig. S10). Furthermore, in accordance with our observation that blocking of acidification by bafilomycin allowed faster germination of *A. terreus*, *A. terreus wA* germinated and formed filaments within the first 12 h of incubation within macrophages (Fig. 7E). Since the phagocytosis rate of *A. terreus wA* was comparable to *A. terreus* wild type, the increased cell damage was unlikely to be mediated by increased numbers of non-phagocytosed *A. terreus wA* conidia germinating extracellularly. However, cell damage and germination of *A. terreus wA* was further enhanced by addition of bafilomycin (Fig. 7F and G). Thus, the observed difference in cytotoxicity of *A. terreus wA* compared to *A. fumigatus* could be due to a lower efficiency of naphthopyrone in blocking of phagolysosome acidification. In summary, these data demonstrate that the expression of *wA* and the production of naphthopyrone was sufficient to significantly alter the interaction of *A. terreus* with macrophages, resulting in a phenotype displaying some characteristics of *A. fumigatus* interaction with macrophages.

### *A. terreus wA* displays enhanced virulence in a mouse model of aspergillosis

**Figure 8:**
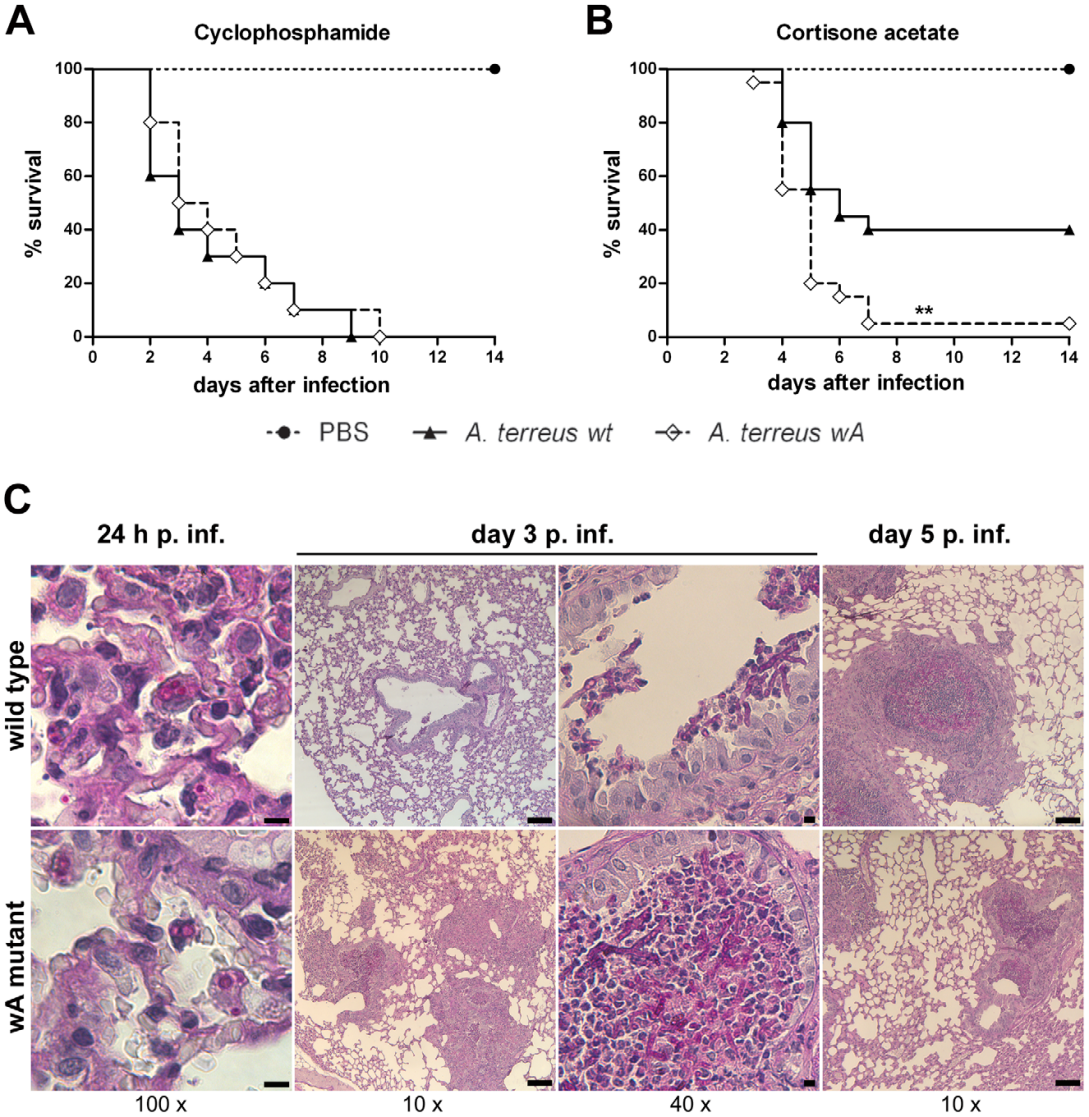
Outcome of infection with *A. terreus* and *A. terreus wA* in immunocompromised mice. (A, B) Kaplan-Meyer survival curves analyzed by log rank test. (A) Leukopenic mice immunosuppressed with cyclophosphamide. (B) Mice immunosuppressed with cortisone acetate. (C) Histology 24 h, day 3 and day 5 post infection (p. inf.). Periodic Acid Shiff stain, fungal elements stain pink. Magnifications are indicated below the columns; scale bars represent 100 µm (10×) and 10 µm (40× and 100×). Upper row: *A. terreus* wild type. Lower row: *A. terreus wA*.

Given that wild type *A. terreus* conidia persist within alveolar macrophages of immunocompetent mice *in vivo*, we postulated that the accelerated escape of *A. terreus wA* conidia from macrophages observed *in vitro* could influence virulence *in vivo*. To test this hypothesis, we infected mice rendered either leucopenic by cyclophosphamide, or immunosuppressed by cortisone acetate, intranasally with wild type *A. terreus* or *A. terreus wA*, respectively. Survival curves of leucopenic mice infected with *A. terreus wA* were indistinguishable from animals infected with wild type *A. terreus* (Fig. 8A). Histologically, invasive fungal growth and tissue necrosis were observed in the lungs of moribund mice in both groups (Fig. S11). In contrast, cortisone acetate treated mice infected with *A. terreus wA* succumbed faster and at a significantly higher proportion than mice infected with *A. terreus* wild type (Fig. 8B). Lung pathology was characterized by fungal mycelium surrounded by extensive immune cell infiltrates dominated by neutrophils in moribund animals of either group (Fig. S11). However, immune cell infiltrates on day 3 after infection were more prominent in mice infected with *A. terreus wA* (Fig. 8C). Furthermore, *A. terreus* wild type was isolated from lungs of all surviving animals 14 days after infection and conidia residing within macrophages were found in all lungs at this late time point (Fig. S11). Non-germinated conidia were likewise observed in the lungs of moribund mice infected with *A. terreus* wild type in lung regions not showing cellular infiltrates. Myeloperoxidase, a marker for neutrophils, was >250-fold increased in the lungs of all moribund animals compared to PBS controls (Fig. S11). However, myeloperoxidase in surviving animals infected with *A. terreus* wild type was 10-fold lower than in moribund mice (Fig. S11), consistent with histological analysis, which showed little or no inflammatory cell infiltrates (Fig. S11). These results suggest that either inflammation was less pronounced in surviving animals compared to moribund animals, or that these animals might have survived severe acute inflammation which resolved over time.

## Discussion

Several studies have focused on the interaction of *A. fumigatus* conidia with alveolar macrophages, elucidating mechanisms involved in the uptake of conidia, the fate of conidia within phagolysosomes and the outbreak of hyphae from macrophages [24], [44]. However, it has not been addressed whether mechanisms used by *A. fumigatus* represent common strategies used by all *Aspergillus* spp. able to cause invasive aspergillosis. Although infections with *A. terreus* are less frequently observed than those with *A. fumigatus*, the prognosis for patients is poor [54]. We propose that the outcome of the initial confrontation of inhaled conidia with alveolar macrophages plays a key role in either fungal clearance, persistence, or initiation of lung infections. Furthermore, different survival strategies used by different *Aspergillus* species may lead to different pathological patterns.

Previous studies have shown that the uptake of *A. fumigatus* conidia by alveolar macrophages is a dynamic process and that the number of phagocytosed conidia increases over time [55], [56]. We likewise observed a time dependent increase of *A. terreus* phagocytosis. However, uptake of *A. terreus* conidia was significantly faster than phagocytosis of *A. fumigatus* conidia, especially if resting conidia were used. Rapid complete uptake of *A. terreus* conidia was likewise observed by Green et al. [57] *in vivo*. Phagocytosis kinetics are influenced by two factors: (i) form and size of the particle and (ii) interaction with specific cell surface receptors, e.g. PAMPs and PRRs [58]. *A. terreus* conidia are comparatively smaller than *A. fumigatus* conidia, which might impact phagocytosis. However, we showed that the size difference of resting and swollen *A. terreus* conidia did not affect phagocytosis rates (Fig. S2 and S3). Thus, the difference in the size of conidia is unlikely to fully explain the different phagocytosis rates of *A. fumigatus* and *A. terreus*. Importantly, we observed higher exposure of β-1,3-glucan and galactomannan on the surface of pre-swollen and resting *A. terreus* conidia, in agreement with the microscopical analysis of β-1,3-glucan exposure performed by Deak et al. [59]. This exposure is likely to contribute to or mediate the faster phagocytosis rates of *A. terreus* conidia. However, it should be noted that prolonged pre-incubation of *A. fumigatus* conidia leads to a strong increase of β-1,3-glucan exposure on the surface of early germlings that impacts recognition and uptake by macrophages [22], [23].

Recognition of β-1,3-glucan by macrophages presented on *A. fumigatus* germlings is mediated by dectin-1 [22], [23] while recognition of mannans is more complex and involves a variety of pathogen recognition receptors, including the mannose receptors, TLR-2 and TLR-4 [34], [60]. Our blocking experiments demonstrate that dectin-1 and mannose receptors are the main PRRs mediating phagocytosis of *A. terreus* conidia. In contrast, even the combined blocking of dectin-1 and mannose receptors and simultaneous blocking of the four receptors dectin-1, mannose receptors, TLR-2 and TLR-4 only moderately reduced phagocytosis of *A. fumigatus* conidia. Thus, additional PRRs, for example DC-SIGN (dendritic cell-specific ICAM-3-grabbing nonintegrin) [61], may be required for phagocytosis of *A. fumigatus*, but not *A. terreus* conidia.

As mentioned above, the pre-swelling time used significantly influences PAMP exposure on *A. fumigatus* conidia and longer pre-swelling exposes β-1,3-glucan facilitating interaction with dectin-1 [22]. In resting *A. fumigatus* conidia, immunogenic molecules are masked by the rodlet layer [12]. However, the immunogenic molecules become exposed during germination, thus explaining enhanced phagocytosis of pre-swollen *A. fumigatus* conidia. The higher exposure of β-1,3-glucan and galactomannan on the surface of resting *A. terreus* conidia and the high phagocytosis rate raises the question to which extend a similar masking rodlet layer is present on *A. terreus*.

One function of alveolar macrophages is the elimination of inhaled pathogens by intracellular killing after phagocytosis. We determined killing of *A. fumigatus* and *A. terreus* conidia by two independent methods: CFU plating and analysis on the single-conidia level using a metabolic dye [62]. Results obtained with either method showed significantly higher survival rates of *A. terreus* compared to *A. fumigatus*. This effect was independent from the fungal strain and type of macrophage used. In contrast, Perkhofer et al. [63] did not observe these striking differences. In their study, survival was analyzed after a short two hour co-incubation using resting instead of pre-swollen conidia. Since the initial swelling process is essential for conidia inactivation [64], this experimental setup might have masked the differences between both species.

Besides an impact of macrophage NADPH oxidase in killing of *A. fumigatus* conidia [64], microbial inactivation by macrophages has been shown to be associated with acidification of the phagolysosome [24], [25]. Similar to other pathogenic fungi [16], *A. fumigatus* is able to inhibit phagolysosome acidification at least to a certain extent [25]. Surprisingly, despite the high survival rates of phagocytosed conidia, phagolysosomes containing *A. terreus* acidified frequently, independent of the macrophage type and *A. terreus* strain used, suggesting that (i) *A. terreus* lacks the ability to block acidification and (ii) *A. terreus* is not inactivated within mature acidic phagolysosomes. Supporting the later, we found that low pH does not impact survival of *A. terreus* conidia under nutrient limited conditions, although acidic pH inhibits germination. In contrast, survival of *A. fumigatus* conidia was significantly impaired at low environmental pH, highlighting the need of *A. fumigatus* to inhibit phagolysosome acidification. However, it should be noted that survival within acidified phagolysosomes does not only depend on pH resistance, but also on the resistance against microbicidal enzymes and compounds, which display highest activity at low pH.

Since conidia are more resistant to damage by adverse environmental conditions than hyphae [65], the delayed germination of *A. terreus* at low pH might enhance persistence in phagolysosomes. In contrast, rapid germination due to inhibition of acidification allows *A. fumigatus* to escape from macrophages, but also renders the fungal cells more vulnerable to antifungal host molecules. This might explain the observed inactivation of a significant proportion of *A. fumigatus* conidia during the time course of co-incubation.

Alveolar macrophages have been shown to efficiently clear *A. fumigatus* conidia in immunocompetent mice [64], resulting in steadily decreasing fungal burdens [66], [67]. In contrast, we found *A. terreus* conidia to persist in stable numbers in the lungs of immunocompetent mice. Histologically, we observed conidia within alveolar macrophages, suggesting that persistence occurs within these immune cells. Although several cytokines and myeloperoxidase significantly increased in immunocompetent mice infected with *A. terreus*, the absolute increase was lower than described for *A. fumigatus* by others [66], [67]. Furthermore, immunocompetent mice infected with *A. fumigatus* rapidly recruit immune cells to the site of infection, peaking two days after infection and resolving towards day 4 [67]. In comparison, immune cell recruitment after *A. terreus* infection was delayed. These results suggest that the differences in macrophage interaction observed for the two fungal species *in vitro* have significant consequences for fungal survival and inflammation *in vivo*. It appears that divergent strategies have evolved within pathogenic *Aspergillus* species: While *A. terreus* survives within acidified phagolysosomes of macrophages, *A. fumigatus* interferes with phagolysosome maturation, germinates and eventually escapes from macrophages. This hints to different strategies to evade the recognition by the immune system. Similarly, different strategies for phagolysosomal survival have also been described for other pathogenic fungi [16]. For example, *Candida albicans* [68] and *Histoplasma capsulatum* [69] also inhibit acidification of the phagolysosome, whereas *Crytococcus neoformans* resides and replicates within acidified phagolysosomes [70], [71].

It has been shown that the ability of *A. fumigatus* to prevent phagolysosome maturation depends on the presence of a functional *pksP* gene. PksP is essential for the synthesis of DHN-melanin and deletion of *pksP* results in white colored conidia [72]. In agreement with the proposed role of DHN-melanin in preventing acidification [73], an *A. fumigatus pksP* deletion mutant cannot inhibit acidification of phagolysosomes and shows altered conidia morphology [47] with significantly increased β-1,3-glucan exposure [23]. Thus, this mutant is killed to a higher rate by macrophages [25]. *A. terreus* lacks this specific *pksP* gene and, although the origin of its pigment is unknown, its conidia color appears not to be DHN-melanin related. Expression of the *pksP* homologue *wA* from *A. nidulans* in *A. terreus*, which leads to the production of the heptaketide naphthopyrone YWA1 [52], was sufficient to prevent acidification of phagolysosomes for the majority of intracellular conidia. This result emphasizes the role of naphthopyrone derivatives in the interaction of *Aspergillus* species with macrophages. However, the reduced acidification by *A. terreus wA* conidia was not sufficient to increase fungal cytotoxicity to the level of *A. fumigatus* and addition of bafilomycin still enhanced cytotoxicity of *A. terreus wA* mutants. This suggests that (i) naphthopyrone might be less efficient than DHN-melanin in interfering with phagolysosome acidification, (ii) that the expression level of *wA* in *A. terreus wA* does not reach the level of DHN-melanin in *A. fumigatus* or (iii) that additional, yet unidentified, *A. fumigatus* factors absent in *A. terreus* interfere with phagolysosome maturation. Furthermore, it should be noted that the lack of naphthopyrone production alone does not sufficiently explain the difference between *A. terreus* and *A. fumigatus* in the interaction with macrophages. For example the mechanisms leading to the high survival rates of *A. terreus* in acidified phagolysosomes remain to be determined.

It is tempting to speculate that the lack of a *pksP* homologue in *A. terreus* contributes to the lower incidence of *A. terreus* infections compared to *A. fumigatus*, as the impact of *pksP* on infection *in vivo* has been demonstrated for *A. fumigatus* [72]. However, the distribution of *pksP* homologues within aspergilli does not directly correlate with virulence in mouse models: Both, *A. nidulans* and *A. niger* possess *pksP* homologues and show a green or black spore coloration. Nonetheless, only *A. niger* is highly virulent in mice [74] while *A. nidulans* [75], [76] shows a more attenuated phenotype. Therefore, we investigated the virulence of the *wA* expressing *A. terreus* mutant in leucopenic and cortisone acetate treated mice.

For *A. fumigatus* it has been shown that the outcome of infection in leucopenic animals is mainly determined by fungal growth [77], due to the absence of the recruited cellular immune response [78]. At the inoculum used, resident macrophages are not able to prevent mycelia formation. In agreement, no differences in virulence were observed between *A. terreus* wild type and *A. terreus wA* in this model, suggesting that *A. terreus wA* does not have a general growth advantage within the lung.

Corticosteroid treatment does not affect phagocytosis of *A. fumigatus* by alveolar macrophages but impairs the ability of macrophages to kill ingested conidia [64], [79]. Thus, due to the low frequency of germination of *A. terreus* wild-type conidia after phagocytosis by macrophages and the accelerated escape of *A. terreus wA* conidia, we expected significant differences in the outcome of infection in the corticosteroid-based model. In agreement, animals infected with *A. terreus wA* succumbed to infection faster and to a significantly higher rate. As persisting, non-germinated conidia were found in all lungs of mice surviving *A. terreus* wild type infection, the difference in virulence could be explained by a comparatively lower induction of inflammation in surviving mice, due to a lower percentage of conidia which germinate. The assumption of low germination rates and persistence is also in agreement with the isolation of fungal burdens from immunocompetent mice. After infection with 1×10^7^ *A. terreus* wild type conidia, at least 5×10^5^ conidia were isolated five days after infection of immunocompetent mice. In contrast, from immunocompetent mice challenged with 2.5×10^7^ *A. fumigatus* conidia only 500 CFUs were isolated four days after infection [67]. Thus, fast escape from macrophages (*A. terreus wA* and *A. fumigatus*) appears to cause increased virulence in corticosteroid treated animals, whereas a “sit and wait” strategy (*A. terreus*) allows long-term persistence, especially in immunocompetent hosts. However, we can not exclude that expression of *wA* in *A. terreus* affects general metabolism or expression of virulence-associated factors and, therefore, might indirectly affect virulence. It should also be noted that, in general, the virulence potential of aspergilli is the result of a multitude of factors which contribute to survival and growth within the host. Thus, it is likely that in addition to pigment production other factors influence the virulence potential of different *Aspergillus* species.

Interestingly, *A. terreus* infections are accompanied by high dissemination rates [5], [11]. We speculate that dissemination might be due to persistence in macrophages. By hiding from immune responses, *A. terreus* might use macrophages as a vehicle to reach secondary organs, establishing disease under severe immunosuppression. Macrophages as vehicle for dissemination have been described for other microbes [80], [81], [82].

It should be noted that *A. terreus* also produces accessory conidia, which differ from the phialidic conidia used in this study in surface structure, cell wall composition and germination kinetics [59]. Furthermore, accessory conidia have been shown to expose higher levels of surface β-glucan, associated with a stronger immune response compared to phialidic conidia *in vitro* and *in vivo* [36]. It thus appears likely that the interaction of accessory conidia with macrophages might differ significantly from phialidic conidia; however, phialidic conidia are the commonly produced distributable spores and thus represent the primary infectious structures of *A. terreus*.

In summary, *A. fumigatus* and *A. terreus* demonstrate striking differences in the interaction with macrophages. *A. fumigatus* prevents phagolysosome acidification due to the presence of DHN-melanin, germinates and escapes from macrophages, leading to establishment of invasive disease in immunocompromised hosts. In contrast, *A. terreus* is rapidly recognized and phagocytosed by macrophages and persists without germination in acidified phagolysosomes. It appears possible that *A. terreus* conidia inhaled by patients prior to severe immunosuppression persist in a resting, but viable state. Thus, development of disease by *A. terreus* might progress more slowly and require a stronger immunosuppression regimen than *A. fumigatus* but does not necessarily require a *de novo* infection due to long-term persistence in macrophages.

## Materials and Methods

### Ethics statement

The use of human primary cells in this study was conducted according to the principles expressed in the Declaration of Helsinki. All protocols used in this study were approved by the local ethics committee of the University Hospital Jena (Permit Number: 2207-01/08; studies on the interaction of human pathogenic fungi with blood and blood components). Written informed consent was provided by all study participants.

All animals used in this study were housed in groups of five in individually ventilated cages and were cared for in strict accordance to the principles outlined in the European Convention for the Protection of Vertebrate Animals Used for Experimental and Other Scientific Purposes (http://conventions.coe.int/Treaty/en/Treaties/Html/123.htm). All animal experiments and protocols were in compliance with the German Animal Welfare Act. All protocols were approved by the responsible Federal State authority (Thüringer Landesamtes für Lebensmittelsicherheit und Verbraucherschutz) and its ethics committee (Permit Number: 03-012/10). All efforts were made to minimize suffering. Animals were clinically monitored at least twice daily by a veterinarian and humanely sacrificed if moribund (defined by lethargy, dyspnoea, hypothermia and weight loss).

### Fungal strains and growth conditions

All experiments were performed with *A. terreus* isolate SBUG844 (HKI strain collection, Jena, Germany, [83]), and *A. fumigatus* isolate CBS 144.89 (CBS, Utrecht, Netherlands). For selected experiments, *A. terreus* A1156 (NIH 2624, Fungal genetics stock center, Kansas; USA) and the clinical isolate T9 (kindly provided by Cornelia Lass-Flörl, Innsbruck, Austria), as well as *A. fumigatus* Af293 (ATCC, Manassas, USA) and ATCC 46645 (ATCC, Manassas, USA) were used. Strains were grown on malt extract agar slants for 7 days at room temperature. Conidia were harvested in 5 ml sterile phosphate buffered saline (PBS, PAA Laboratories, Coelbe, Germany) and filtered at least once through a 40 µm cell-strainer (BD, Heidelberg, Germany). If not indicated otherwise, pre-swollen conidia were obtained after 7 h (*A. terreus*) or 3.5 h (*A. fumigatus*) incubation in sterile RPMI 1640 (PAA Laboratories) supplemented with 10% heat-inactivated fetal bovine serum (FBS, PAA Laboratories) at 37°C and 20 rpm shaking [64]. Heat inactivation was facilitated by exposing the FBS 20 min to 56°C. After incubation, cells were centrifuged, washed three times with sterile PBS and again filtered through a 40 µm cell-strainer.

For phagocytosis and killing assays, conidia were pre-labeled with FITC after swelling as described elsewhere [84]. Conidia were counted with a Neubauer counting chamber and diluted in sterile PBS or RPMI with 10% FBS to give the desired concentration. Absence of clumping was confirmed in all conidia preparations by microscopy immediately prior to use.

### Macrophage cell lines

The alveolar macrophage cell line MH-S, derived from the lung of a Balb/C mouse and immortalized by simian virus SV40 transfection (ATCC, Mannassas, USA), was routinely kept in RPMI 1640 supplemented with 10% heat-inactivated FBS. These alveolar macrophages display the morphologic features and functions of normal, mature alveolar macrophages which were derived from bronchioalveolar lavages [85]. The peritoneal macrophage-like cell line J774.A1 (DSMZ, Braunschweig, Germany) derived from an ascites of a Balb/C mouse and was kept in Dulbecco's modified Eagle's medium (DMEM, PAA Laboratories) with 10% heat-inactivated FBS. Both cell lines were maintained and propagated at 37°C and 5% CO_2_ atmosphere. These cells show the morphologic, cytotoxic and adherence properties of primary peritoneal macrophages [86].

For interaction experiments, macrophages were used between passages 10 and 20 and seeded at a concentration of 2×10^5^ per well in 8 well chamber slides (Nunc, Roskilde, Denmark) 24 h prior to co-incubation with conidia, leading to a confluent monolayer at the time of experimentation.

### Primary macrophages

Human peripheral blood mononuclear cells (PBMCs) were isolated with Histopaque-1077 (Sigma-Aldrich) density centrifugation from buffy coats donated by healthy volunteers. To differentiate PBMC into monocyte-derived macrophages (MDMs), 2×10^7^ PBMCs were plated in RPMI 1640 media (with L-glutamine and 25 mM HEPES; PAA Laboratories, Inc.) containing 10% heat-inactivated FBS (Gibco, Invitrogen) in cell culture dishes. Ten ng/ml human M-CSF (ImmunoTools, Friesoythe, Germany) were added to the cultures to induce differentiation into macrophages. After 5 days at 5% CO_2_ and 37°C, non-adherent cells were removed and purity of MDMs was determined by staining with an anti-CD14 antibody and flow cytometry. The percentage of CD14-positive cells was ≥80%. All experiments were performed with cells isolated from at least three different donors.

For infection experiments, adherent MDMs were detached with 50 mM EDTA and plated in flat-bottom 96 or 24 well plates containing RPMI 1640 with 10% FBS to give a final concentration of approximately 5×10^4^ cells/well or 2×10^5^ cells/well, respectively. For microscopic analyses cells were allowed to adhere over night to 8-well chamber slides prior to infection.

Murine alveolar macrophages and bone marrow-derived macrophages were isolated from healthy, specific pathogen free eight to ten weeks old CD-1 mice. In brief, mice were euthanized, and the trachea was prepared in situ. 1 ml of sterile PBS was instilled *via* the trachea into the lung and the procedure was repeated twice. The bronchoalveolar lavage fluid of ten mice was pooled and centrifuged for 10 min at 300×g. The pellet was resuspended in RPMI 1640 supplemented with 10% FBS and 1% Penicillin/Streptomycin (PAA Laboratories). Cells were plated in chamber slides at a density of 2×10^5^ cells/well and used one day after inoculation. For bone marrow-derived macrophages, the femurs were aseptically opened and the bone marrow was removed by washing with 2 ml of sterile, ice-cold PBS. After washing, the cell suspension was vortexed vigorously. The cells were pelleted by centrifugation for 10 min at 300×g, resuspended in RPMI 1640 supplemented with 10% FBS and filtered through a 40 µm cell strainer (BD). Cells were seeded at 1×10^6^ cells/well in 6-well polystyrene plates containing RPMI 1640 supplemented with 10% FBS, 1% Penicillin/Streptomycin and 10 ng/ml murine M-CSF (ImmunoTools, Friesoythe, Germany). After five days of incubation at 37°C and 5% CO_2_, non-adherent cells were removed by washing three times with sterile PBS. Fresh RPMI 1640 supplemented with 10% FBS was added prior to challenge with conidia.

### Phagocytosis assay

Phagocytosis assays were performed as described previously [24] with some modifications. Macrophage cell lines were plated in 8-well chamber slides in RPMI or DMEM, depending on the cell line, supplemented with 10% heat-inactivated FBS. Cells were infected with an MOI of 0.1 (1 conidium per 10 macrophages). After brief centrifugation, slides were further incubated for up to 12 h. At the end of each incubation period the assay medium was removed and cells were fixed directly with Histofix® (Roth, Karlsruhe, Germany) for 10 min at room temperature (RT). After washing with 0.05 M NH_4_Cl, cells were incubated for 10 min in 0.05 M NH_4_Cl dissolved in PBS.

Prior to addition of antibodies, cells were blocked for 30 min at RT with 1% bovine serum albumin (BSA; Sigma). After blocking, slides were incubated for 30 min at RT with a specific rabbit anti-conidia antiserum at a dilution of 1∶50 (kindly provided by Frank Ebel). For fluorescence detection, slides were incubated with a specific anti-rabbit Texas Red conjugated antibody (Invitrogen, Groningen, Germany) diluted 1∶1000 in blocking solution for 30 min at room temperature in the dark.

Slides were washed three times with PBS and analyzed by microscopy. Phagocytosed conidia only showed a bright green fluorescence (deriving from FITC-labeling) while extracellular conidia additionally showed a red signal. Phagocytosis rate in percent was determined as the number of ingested conidia per 1000 conidia counted. Three individual wells were evaluated per slide, strain and time point and experiments were performed in biological triplicates.

### Receptor blocking assay

For dectin-1 blocking, macrophages were incubated for 10 min prior to infection on ice in culture medium containing 10% heat-inactivated FBS with 2 mg/ml laminarin from *Laminaria digitata* (Sigma). Mannose receptors were blocked by addition of 2 mg/ml mannan from *Saccharomyces cerevisiae* (Sigma) to the culture medium 15 min prior to infection [87]. Blocking of TLR2 and TLR4 was performed over night at 37°C before infection by adding 15 µg/ml of specific antibodies (anti-TLR2: affinity purified anti-mouse Toll-like receptor 2 (CD282, TLR2), clone 6C2, eBioscience, Frankfurt, Germany; anti-TLR4: affinity purified anti-mouse Toll-like receptor 4 (TLR4/MD-2), clone MTS510, eBioscience) [88]. Prior to infection, the medium containing blocking reagents was removed, the cells were washed three times with sterile PBS (PAA) and then infected with conidia in medium containing 10% heat-inactivated FBS. After infection with pre-swollen conidia the standard phagocytosis assay was performed as described above.

### Flow cytometric analysis of conidia

Flow cytometry was used to quantify the β-1,3-glucan and galactomannan exposure of *A. terreus* and *A. fumigatus* on resting and pre-swollen conidia by using specific antibodies (mouse IgG2b anti β-1,3-Glucan [89]; mouse IgM anti-galactomannan [90]; isotype controls: mouse IgG2b/IgM; eBioscience). Resting and pre-swollen conidia were investigated in parallel. Resting and pre-swollen conidia were incubated for 1 h in Histofix® (Roth) at RT, then for 1 h in NH_4_Cl (0.05 M) and finally washed three times with PBS. To inhibit unspecific binding of primary antibodies, conidia were incubated in 1% BSA for 1 h at RT. After washing with PBS, conidia were incubated at 4°C over night on a vertical rotator with primary antibodies diluted in blocking solution. Following three times washing with PBS a FITC-labeled goat anti mouse IgG/IgM antibody (Invitrogen) was applied, and conidia were incubated for 3 h at RT in the dark. After additional five washings with PBS, measurement was performed in flow cytometry buffer (BD) on a Becton Dickinson LSRII. 20,000 events were counted. At least three biological replicates were analyzed. Data were further analyzed with the FlowJo software (Tree Star, Ashland, USA).

### Killing assay

To quantify killing of conidia by macrophages, co-incubations of macrophages and pre-swollen conidia (performed as described for phagocytosis assay) were stained with the FUN-1 dye (Invitrogen) [62] according to the manufacturers guidelines, resulting in distinct fluorescence patterns. Living conidia showed green to yellowish cytoplasm with a bright red vacuole, while dead conidia showed a yellow cytoplasm (Axio Imager M1, Filter Set 01; BP 365/12, FT 395, LP 397, Zeiss, Jena, Germany). Viability of conidia without macrophages was assessed in each experiment. Both *Aspergillus* species were analyzed in parallel. Killing rate was determined from the number of inactivated/dead conidia per number of conidia investigated. In each experiment 1000 conidia per strain were evaluated. The experiments were performed in three biological replications each containing three technical replicates. Alternatively, killing was quantified as CFUs. Here, either primary murine BMDMs or the alveolar macrophage cell line MH-S were plated at a density of 1×10^6^ cells/well in 6 well polystyrene plates one night prior to infection. Conidia of the indicated strains were added at an MOI of 0.1 and incubated for 3 h and 10 h. After incubation, macrophages were lysed with ice-cold sterile deionised water for 15 min. After lysis the cells were scraped from the bottom of the well with a rubber policemen and the suspension was diluted for plating. Serial dilutions were plated on malt extract agar plates and incubated for at least 36 h at 37°C. The percentage of survival was calculated with reference to control conidia incubated in the absence of cells.

### Macrophage monolayer damage assay

Damage to macrophages after co-incubation with pre-swollen conidia (performed as described for phagocytosis assay) was determined as the release of lactate dehydrogenase (LDH) into the medium using a Cytotoxicity Kit-LDH (Roche, Mannheim, Germany). The assays were performed according to the manufacturer's instructions and the measurements from three biological replications containing technical triplicates were evaluated. To measure cell damage, the following calculation was used: 100× LDH release (infected macrophages−uninfected macrophages−conidia only)/(100% lysed macrophages−uninfected macrophages) = relative cytotoxicity (%). Uninfected macrophages, conidia only and 100% macrophage lysis (achieved by adding 0.2% Triton X-100, Applichem) served as controls and were determined individually for each treatment and incubation period.

### Transmission electron microscopy

For TEM, alveolar macrophages were grown in 24 well plates with a polystyrene bottom. *A. terreus* was added at a MOI of 1. Eight and 24 h post infection, samples were washed 3 times with PBS to remove non-adherent cells. Samples were directly fixed with Karnovsky fixative (3% paraformaldehyde, 3.6% glutaraldehyde, pH 7.2) for 24 h. Post-fixation was based on 1.0% osmium tetroxide containing 1.5% K-ferrocyanide in 0.1 M cacodylate buffer for 2 h.

Following standard methods, blocks were embedded in glycide ether and cut using an ultra microtome (Ultracut, Reichert, Vienna, Austria). Ultra-thin sections (30 nm) were mounted on copper grids and analyzed using a Zeiss LIBRA 120 transmission electron microscope (Carl Zeiss,Oberkochen, Germany) operating at 120 kV.

### pH resistance of conidia

1× 10^6^ pre-swollen conidia were incubated for up to seven days at 37°C and 220 rpm in 1 ml 0.1 M sodium phosphate buffer supplemented with 0.3 M potassium chloride and adjusted to the desired pH. From different time points, suspensions were analyzed microscopically and serial dilutions were plated in triplicate on malt extract agar plates (Applichem). Additionally, the pH of the buffer was measured to confirm that the suspension maintained at the desired pH. CFU were counted after 24 h incubation at 37°C. Experiments were performed in triplicates. Survival at pH 7 was set as 100%, and the relative survival compared to pH 7 was determined. For microscopical analysis of the germination speed at different pH values, resting conidia were incubated for 8 h at 37°C and 5% CO_2_ in *Aspergillus* minimal medium [80] with the pH adjusted to the desired value.

### Inhibition of macrophage v-ATPase

To inhibit phagolysosome acidification, bafilomycin A1 (Sigma) was added to macrophage monolayers to a final concentration of 25 nM 10 min prior to infection. After 24 h the relative cytotoxicity was determined by LDH release as described above. Cells treated with bafilomycin in the absence of conidia served as controls.

### Fluorescence microscopy of phagolysosome maturation

Phagolysosome maturation was determined from co-incubations with pre-swollen conidia in 8-well chamber slides. For immunofluorescence analysis, cells were fixed after 3 h and 8 h of co-incubation, permeabilized for 15 min with 0.1% Triton X-100 (Applichem) and blocked for 30 min with Image IT FX signal enhancer (Invitrogen). After incubation, samples were washed with PBS and either incubated for 1 h at RT with the primary antibody against Lamp-1 (rat anti-mouse Lamp-1 clone 1D4B Alexa Fluor 647; Santa Cruz Biotechnology, Heidelberg, Germany; 1∶100 diluted in 1% BSA); or with a Cathepsin D specific antibody (rabbit anti-mouse Cathepsin D antibody clone EPR3057Y; Epitomics Inc., Burlingame, United States; 1∶1000 diluted in 1% BSA). For immunostaining against Cathepsin D an additional 1 h incubation step at RT with a monoclonal goat anti-rabbit Dylight 549 conjugated antibody (Rockland Immunochemicals, Gilbertsville, United States) diluted 1∶5000 in blocking solution was performed. For investigating the acidification of the phagolysosome, cells were pre-incubated 2 h prior to infection at 37°C with Lysotracker DND99 (Invitrogen) diluted 1∶5000 in RPMI supplemented with 10% FBS. After co-incubation for 3 h and 8 h with conidia, cells were fixed as described above, and investigated under a Zeiss LSM 710 microscope (Carl Zeiss, Jena, Germany).

### Murine infection models

Female specific-pathogen-free inbred Balb/C mice (6–8 weeks old, 18 to 20 g) were used in the studies for the leucopenic model, while female specific-pathogen-free outbred CD-1 (6–8 weeks old, 18 to 22 g) were used in the corticosteroid model (Charles River, Germany).

For the leucopenic infection model, animals were immunosuppressed by intraperitoneal (i.p.) injections of cyclophosphamide (140 mg/kg; Sigma, Germany) on days −4, −1, 2, 5, 8, 11 and an additional dose of cortisone acetate (200 mg/kg, Sigma, Germany) subcutaneously (s.c.) on day −1. For the corticosteroid based model, i.p. injections of cortisone acetate (25 mg/mouse) were applied on days −3 and 0 prior to infection.

The effect of the administered drugs was assessed by differential blood cell counts before immunosuppression, before infection and on the day of post mortem analysis.

Studies in immunocompetent mice were carried out by using specific-pathogen-free CD-1 mice untreated prior to infection.

Mice were challenged under general anaesthesia on day 0 intranasally with either 5×10^6^ (cyclophosphamide) or 1×10^7^ (cortisone acetate/immunocompetent) conidia in 20 µl sterile PBS. Control animals received 20 µl of sterile PBS. For general anaesthesia a mixture of Medetomidin (0.5 mg/kg, Fort Dodge Veterinär GmbH, Würselen, Germeany), Midazolam (5 mg/kg, Roche, Mannheim, Germany) and Fentanyl (0.05 mg/kg, Janssen-Cilag, Neuss, Germany) was used and directly antagonized after infection with Antipamezol (2.5 mg/kg, Pfizer GmbH, Berlin, Germany), Flumazenil (0.5 mg/kg, Roche, Mannheim, Germany) and Naloxon (1.2 mg/kg, Hameln Pharma Plus GmbH, Hameln, Germany).

In time course experiments five mice per group were sacrificed 24 h, 72 h and on day 5 after infection. Gross pathological changes were recorded during post mortem analysis. All animals were further analyzed for histological alterations, MPO content in the target organs and cytokines.

### Histology

The right lung of every animal used in this study was fixed in buffered formalin, flatly embedded in paraffin and 5 µm sections were stained with periodic-acid Schiff (PAS) stain using standard protocols. Two distinct sections from every lung were examined qualitatively by light microscopy.

### Generation of *A. terreus* strains heterologously expressing the *A. nidulans wA* gene

In order to generate an *A. terreus* strain capable of producing the *A. nidulans* conidial pigment naphthopyrone, the responsible polyketide synthase gene *wA* from *A. nidulans* (gene accession X65866) with its native promoter and terminator (“*wA*+P+T”; 8131 bp) was amplified from the genomic DNA of the *A. nidulans* reference strain FGSC A4. The Takara PrimeSTAR HS DNA polymerase (MoBiTec, Göttingen, Germany) with oligonucleotides NotI_AN_8209_f1 (5′-GCGGCCGCGTGCATTTGACCAAGCCAC-3′) and NdeI_AN_8209_r15 (5′-CATATGCTGCCAGACACTCTATGACG-3′) was used with the following program: initial denaturation for 4 min at 95°C; 31 cycles with denaturation for 30 s at 95°C, annealing for 30 s at 60°C, elongation for 9 min at 72°C; final elongation for 20 min at 72°C. The gel purified fragment was ligated into pJET1.2/blunt (Fermentas, St. Leon-Rot, Germany) and further subcloned *via Not*I/*Nde*I digestion (Fermentas Fast Digest endonucleases) into plasmid MCS-pJET-pUC19 [83] yielding (*wA*+P+T)-MCS-pJET-pUC19. The plasmid was linearized by *Not*I restriction (Fermentas) and the pyrithiamine resistance (*ptrA*) cassette from plasmid *ptrA*-pJET [91] was inserted. Seven µg of plasmid DNA were used for protoplast transformation of *A. terreus* SBUG844 as described previously [83] using 0.1 µg/ml pyrithiamine hydrobromide (Sigma Aldrich, Hamburg, Germany) as selection marker. A digoxygenin-labeled probe (Roche, Mannheim, Germany) directed against the *wA* gene was generated by PCR amplification with oligonucleotides AN_8209_f9 (5′-GTCTCTACAGCCGACTTTCC-3′) and AN_8209_r11 (5′-GGAAGAGCAATGTCGAGGATC-3′). This probe was used for Southern blot analysis on genomic DNA of transformants to determine the number of genomic integrations. Naphthopyrone production from *wA* transformants was analyzed from 50 mM glucose minimal medium, Czapek Yeast Autolysate (CYA) and YPD agar plates (3 days, 37°C) by comparing the coloration of conidia to that of the parental *A. terreus* strain and an *A. nidulans* strain with deleted *yA* gene [92].

### Detection of naphthopyrone from conidia of *A. terreus wA*

To detect naphthopyrone from conidia of strain *A. terreus wA*, conidia were harvested in water from malt extract agar plates. The suspension was mixed with an equal volume of ethyl acetate and stirred for 10 min using an Ultra Turrax at 20 000 rpm. The ethylacetate phase was removed and extraction was repeated two additional times. Ethyl acetate was evaporated and the resulting dried extract was solved in methanol. The same procedure was repeated for the *A. terreus* wild-type strain. High resolution electrospray ionisation mass spectrometry (HR-ESI MS) was carried out on an Accela UPLC-system (Thermo Scientific) combined with an Exactive mass spectrometer (Thermo Scientific) operating in positive ionisation mode. Separation was carried out on a Betasil C18 column (2.1×150 mm; 3 µm, Thermo Scientific) using water (solvent A) and acetonitrile (solvent B), each containing 0.1% formic acid, as binary solvent system. A flow rate of 250 µL/min and the following gradient was used: 0–1 min = 5% B; 1–16 min = 5%–98% B; 16–19 min = 98% B; 19–20 min = 98%–5% B.

### Myeloperoxidase and cytokine quantification

Organs were homogenized in tissue lysis buffer (200 mM NaCl, 5 mM EDTA, 10 mM Tris, 10% glycerol, 1 mM phenymethylsulfonyl fluorid (PMSF), 1 µg/ml leupeptide and 28 µg/ml aprotinin). Following two centrifugation steps (1500×g, 10 min 4°C) the supernatants were stored at −80°C until use.

Myeloperoxidase and cytokine levels were determined using commercially available murine enzyme-linked immunosorbent assays (ELISA) kits (MPO, Hycult Biotechnology, Uden, Netherlands; cytokine ELISA, eBioscience, Hatfield) according to the manufacturers recommendations.

### Statistical analysis

Statistical analysis was performed using the GraphPad Prism Software (GraphPad Software Inc., La Jolla, United States). Differences were analyzed using the student's paired or unpaired t-test (two-tailed *P* value) or ONE - WAY ANOVA. *P*-values<0.05 were considered as significant (*), and <0.01 as highly significant (**).

## Supporting Information

Figure S1 **Analysis of time dependent germination of** ***A. fumigatus*** **and** ***A. terreus*** **conidia in two different cell culture media.**
(DOC)

Figure S2 **Size determination of resting and swollen conidia by flow cytometry.**
(DOC)

Figure S3 **Survival of different** ***A. fumigatus*** **and** ***A. terreus*** **strains upon co-incubation with MH-S macrophages.**
(DOC)

Figure S4 **Cytotoxicity of different** ***A. fumigatus*** **and** ***A. terreus*** **strains.**
(DOC)

Figure S5 **Phagolysosome acidification in primary macrophages after phagocytosis of** ***A. fumigatus*** **and** ***A. terreus*** **.**
(DOC)

Figure S6 **Phagolysosome acidification in primary macrophages after phagocytosis of different** ***A. fumigatus*** **and** ***A. terreus*** **strains.**
(DOC)

Figure S7 **Influence of bafilomycin on macrophage lysis by different** ***A. fumigatus*** **and** ***A. terreus*** **strains.**
(DOC)

Figure S8 **Conservation of polyketide synthases responsible for conidia coloration among different** ***Aspergillus*** **species.**
(DOC)

Figure S9 **Determination of naphthopyrone from** ***A. terreus wA*** **.**
(DOC)

Figure S10 **Acidification of phagolysosomes in primary macrophages containing** ***A. terreus wA*** **.**
(DOC)

Figure S11 **Histology and quantification of inflammation in mice infected with** ***A. terreus*** **wild type or** ***A. terreus wA*** **.**
(DOC)
