# Supplementary material for: Persistence versus Escape: Aspergillus terreus and Aspergillus fumigatus Employ Different Strategies during Interactions with Macrophages
Source: PLoS One. 2012 Feb 3;7(2):e31223. doi: 10.1371/journal.pone.0031223 (PMC3272006; doi:10.1371/journal.pone.0031223)
Supplement: Figure S1 — Analysis of time dependent germination of A. fumigatus and A. terreus conidia in two different cell culture media. (DOC) [file pone.0031223.s001.doc]

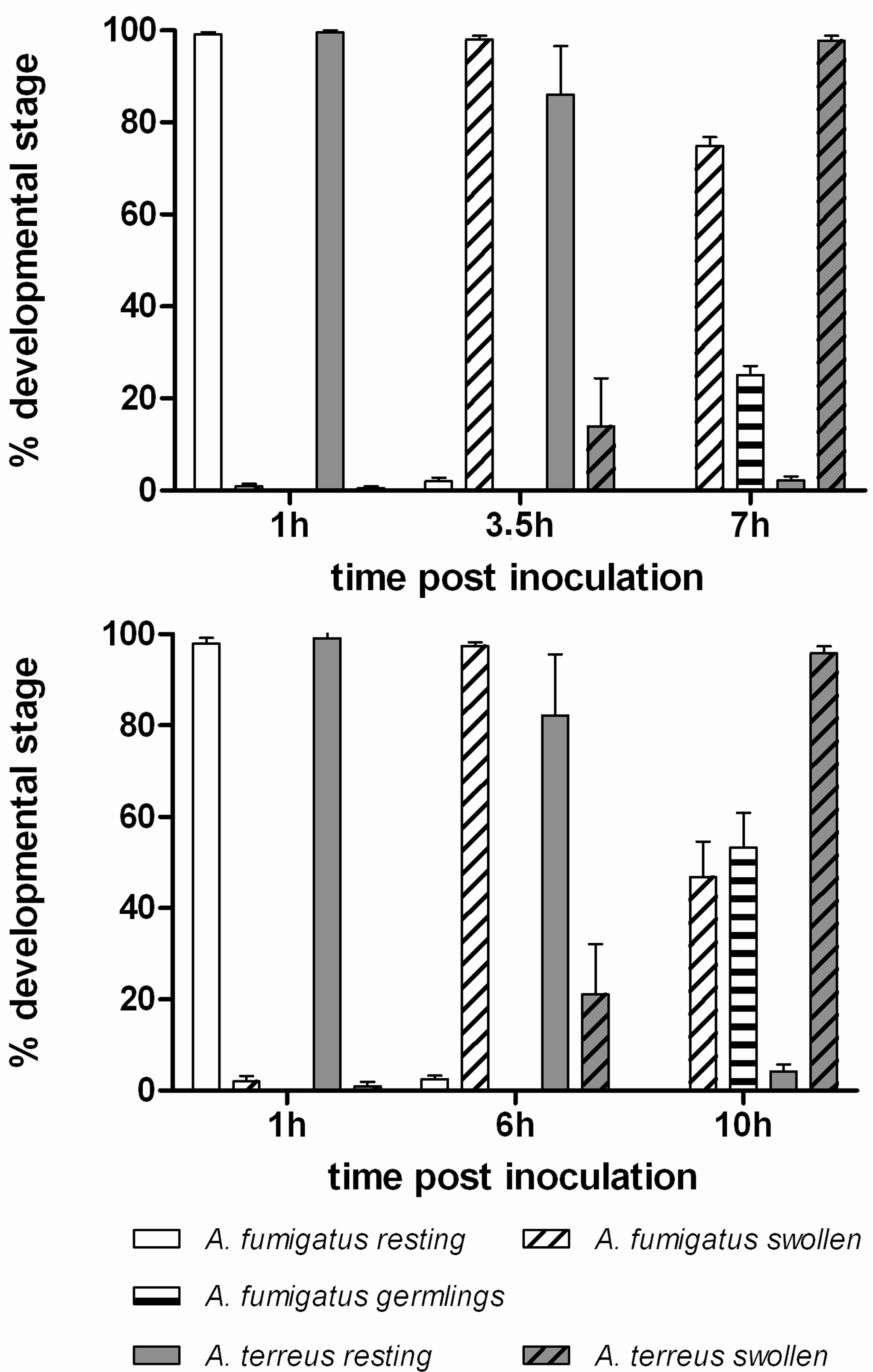


**Figure S1: Analysis of time dependent germination of *A. fumigatus* and *A. terreus* conidia in two different cell culture media.** Resting conidia were used for inoculation and incubated at 37°C. At distinct time points the developmental state was analyzed microscopically from at least 500 conidia. Top: RPMI, bottom: DMEM. In both media *A. fumigatus* swells and produces germlings much faster (≥ 95% swollen conidia after 3.5 h in RPMI and 6 h in DMEM) than *A. terreus* (≥ 95% swollen conidia after 7 h in RPMI and 10 h in DMEM). Data represent means + standard deviation from three independent experiments.
