# Supplementary material for: Persistence versus Escape: Aspergillus terreus and Aspergillus fumigatus Employ Different Strategies during Interactions with Macrophages
Source: PLoS One. 2012 Feb 3;7(2):e31223. doi: 10.1371/journal.pone.0031223 (PMC3272006; doi:10.1371/journal.pone.0031223)
Supplement: Figure S2 — Size determination of resting and swollen conidia by flow cytometry. (DOC) [file pone.0031223.s002.doc]

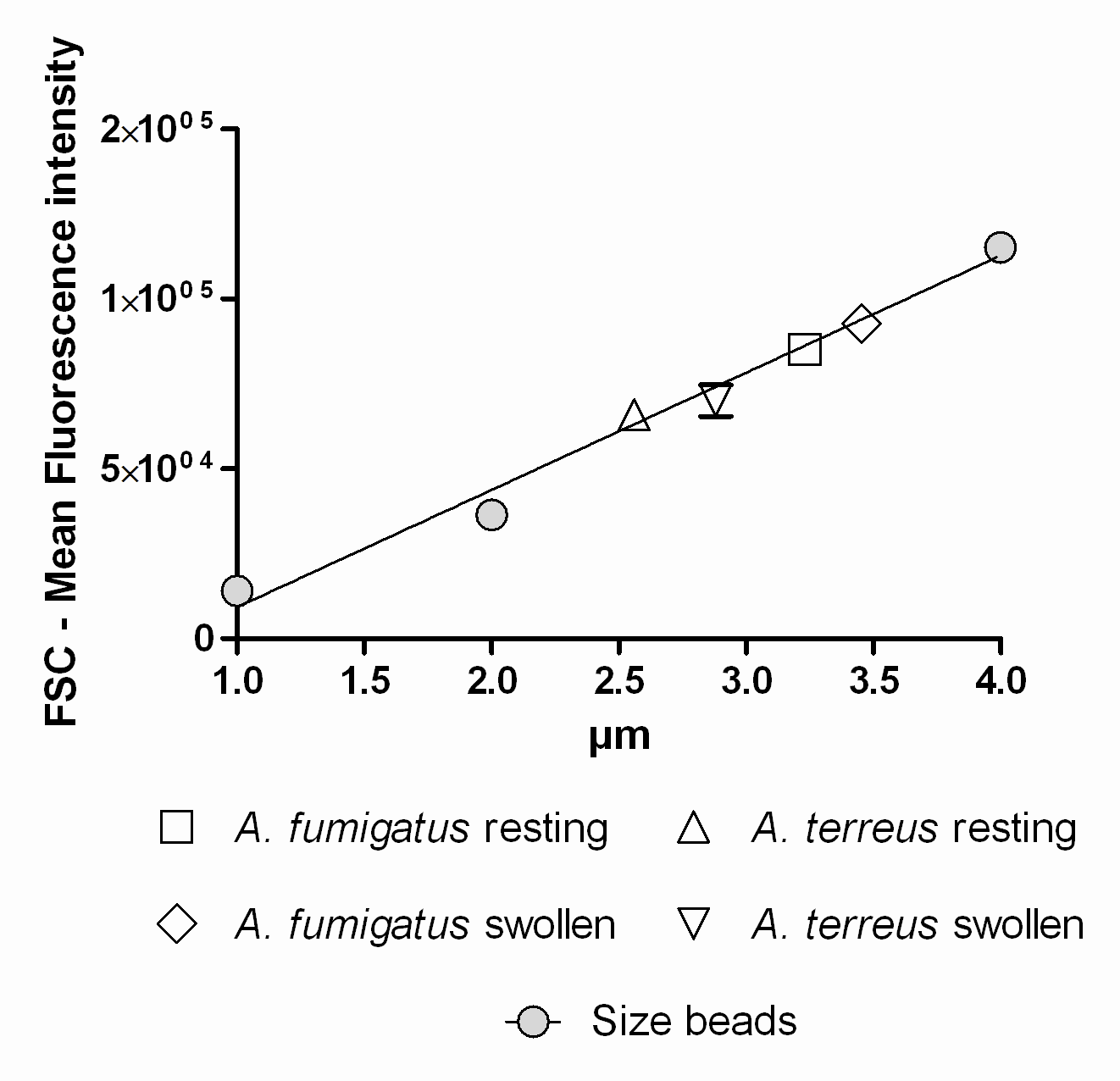


| **Strain** | **Mean Fluorescence Intensity** | **Standard Deviation** |
| --- | --- | --- |
| *A. fumigatus* resting | 85239 | 3089.35 |
| *A. terreus* resting | 65957 | 3128.24 |
| *A. fumigatus* swollen | 92770 | 2310.83 |
| *A. terreus* swollen | 70051 | 4634.38 |

**Figure S2: Size determination of resting and swollen conidia by flow cytometry.** The average size of *A. terreus* and *A. fumigatus* resting and swollen conidia was determined by flow cytometry using calibrated size beads as standard. *A. terreus* resting and swollen conidia possess a smaller diameter than the respective conidia from *A. fumigatus*. Data show the average diameter of 20000 conidia.
