# Supplementary material for: Persistence versus Escape: Aspergillus terreus and Aspergillus fumigatus Employ Different Strategies during Interactions with Macrophages
Source: PLoS One. 2012 Feb 3;7(2):e31223. doi: 10.1371/journal.pone.0031223 (PMC3272006; doi:10.1371/journal.pone.0031223)
Supplement: Figure S3 — Survival of different A. fumigatus and A. terreus strains upon co-incubation with MH-S macrophages. (DOC) [file pone.0031223.s003.doc]

**Figure S3: Survival of different *A. fumigatus* and *A. terreus* strains upon co-incubation with MH-S macrophages.** Survival determined by CFU. Data is shown as mean + SD from three independent experiments; statistical analysis by 1-way ANOVA and Tukey’s multiple comparison test. * P < 0.05; ** P <. 0.01; *** P < 0.001.
