# Supplementary material for: Persistence versus Escape: Aspergillus terreus and Aspergillus fumigatus Employ Different Strategies during Interactions with Macrophages
Source: PLoS One. 2012 Feb 3;7(2):e31223. doi: 10.1371/journal.pone.0031223 (PMC3272006; doi:10.1371/journal.pone.0031223)
Supplement: Figure S4 — Cytotoxicity of different A. fumigatus and A. terreus strains. (DOC) [file pone.0031223.s004.doc]

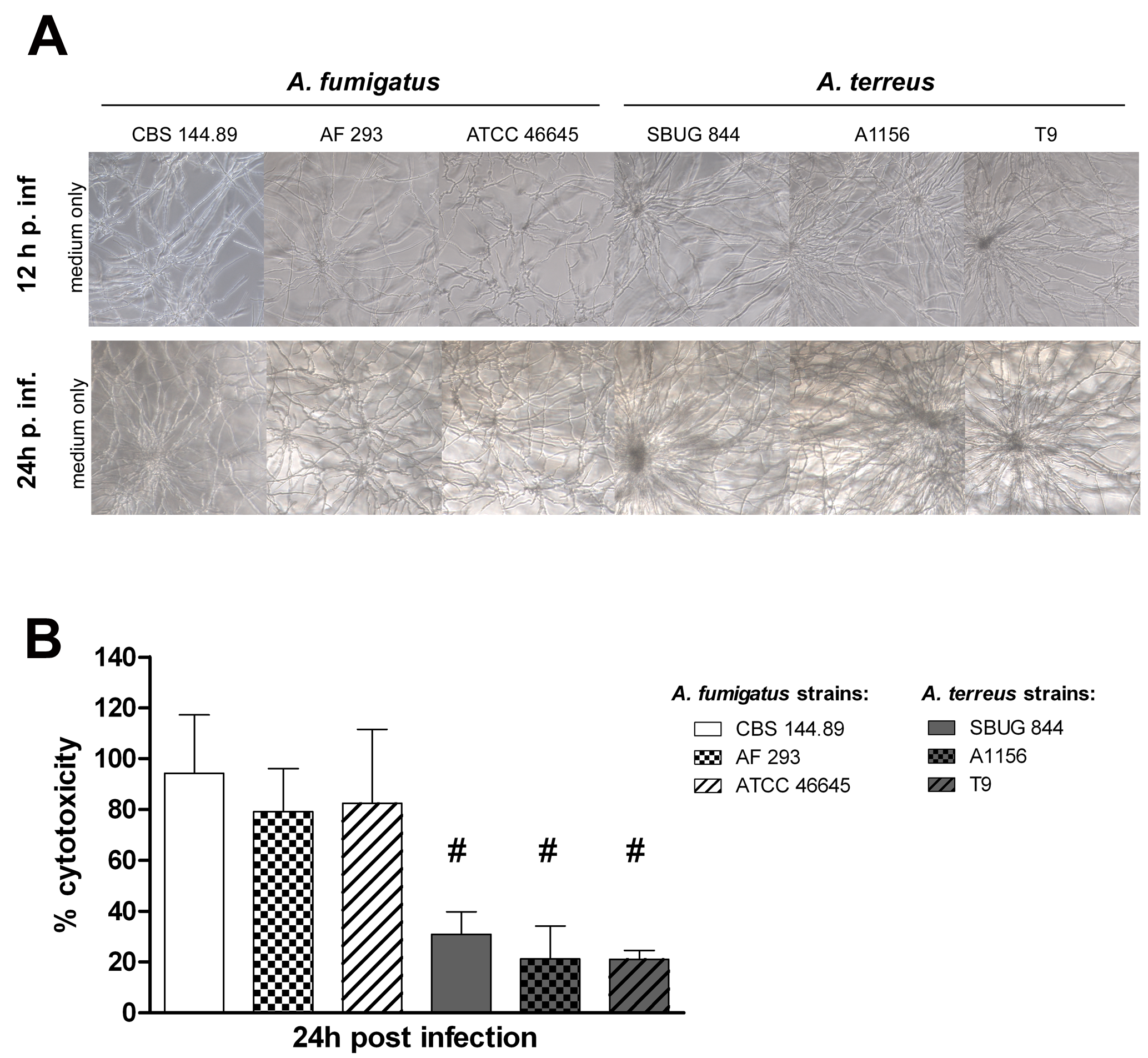


**Figure S4. Cytotoxicity of different *A. fumigatus* and *A. terreus* strains.** (A) Micrograph of growth in cell culture media without macrophages. No gross differences in growth were observed. (B) Relative cytotoxicity of *A. terreus* and *A. fumigatus* conidia to MH-S cells determined by release of lactate dehydrogenase (LDH). Mean values + SD from three independent experiments, statistical analysis was performed by 1-way ANOVA and Tukey’s multiple comparison test. # significantly reduced cytotoxicity (P < 0.05).
