# Supplementary material for: Persistence versus Escape: Aspergillus terreus and Aspergillus fumigatus Employ Different Strategies during Interactions with Macrophages
Source: PLoS One. 2012 Feb 3;7(2):e31223. doi: 10.1371/journal.pone.0031223 (PMC3272006; doi:10.1371/journal.pone.0031223)
Supplement: Figure S7 — Influence of bafilomycin on macrophage lysis by different A. fumigatus and A. terreus strains. (DOC) [file pone.0031223.s007.doc]

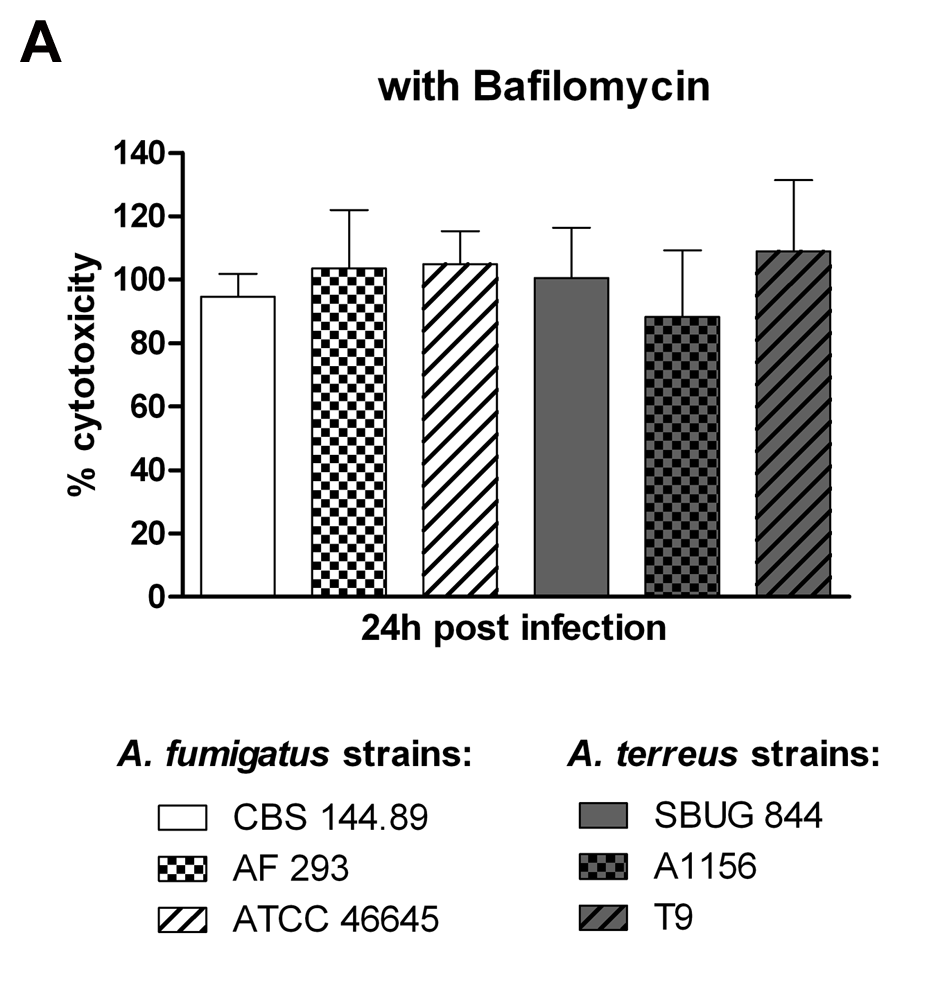


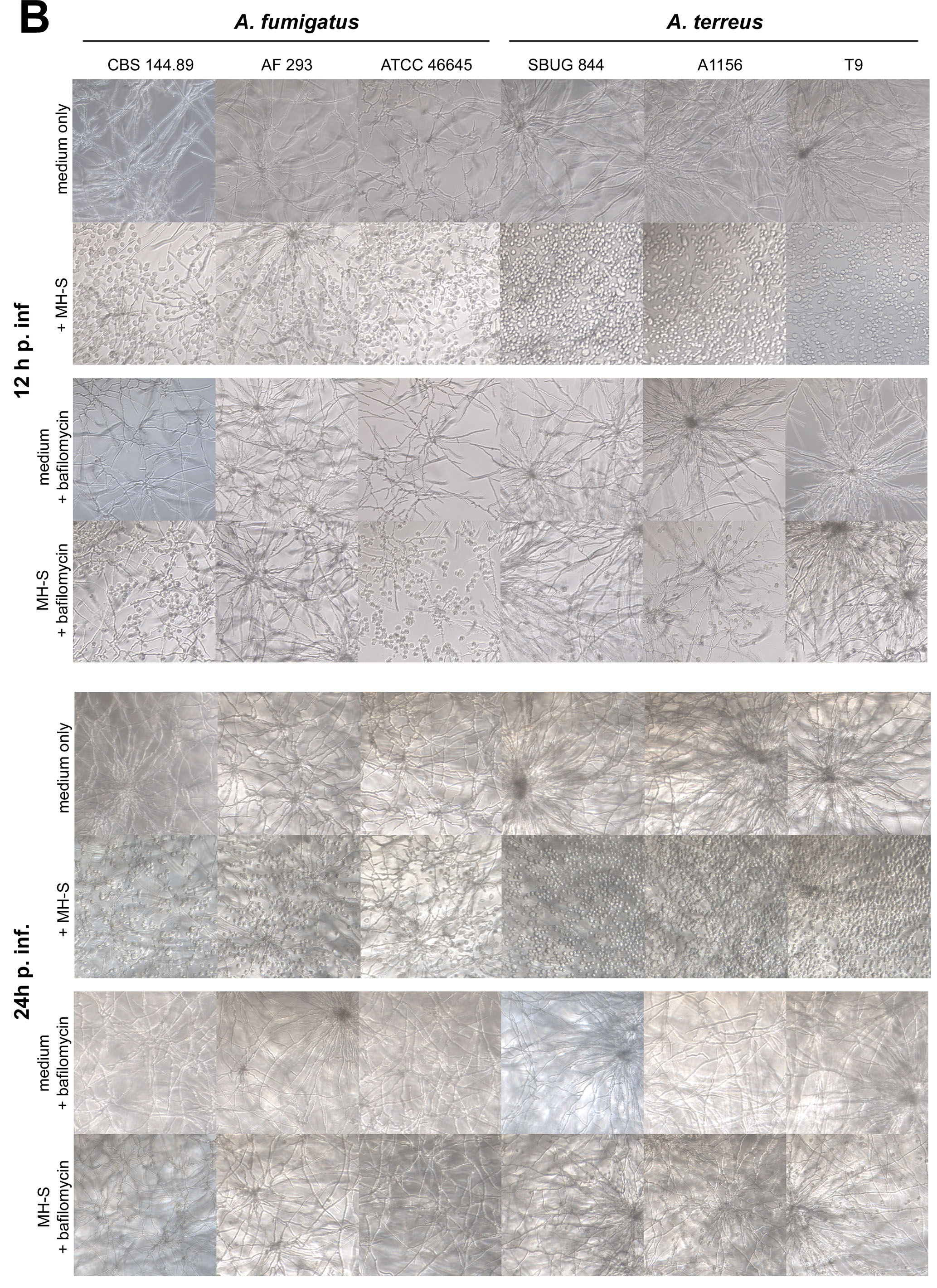


**Figure S7: Influence of bafilomycin on macrophage lysis by different *A. fumigatus* and *A. terreus* strains.** (A) Relative cytotoxicity (LDH release) of *A. terreus* and *A. fumigatus* conidia towards alveolar macrophages (MH-S cells) treated with the v-ATPase blocker bafilomycin (B) Brightfield microscopy of *A. terreus* in co-incubation with medium and macrophages without and with bafilomycin (lower lane) 12 h and 24 h after infection (p. inf.). Bafilomycin treatment allows *A. terreus* to germinate and escape from macrophages.
