# Supplementary material for: Persistence versus Escape: Aspergillus terreus and Aspergillus fumigatus Employ Different Strategies during Interactions with Macrophages
Source: PLoS One. 2012 Feb 3;7(2):e31223. doi: 10.1371/journal.pone.0031223 (PMC3272006; doi:10.1371/journal.pone.0031223)
Supplement: Figure S8 — Conservation of polyketide synthases responsible for conidia coloration among different Aspergillus species. (DOC) [file pone.0031223.s008.doc]

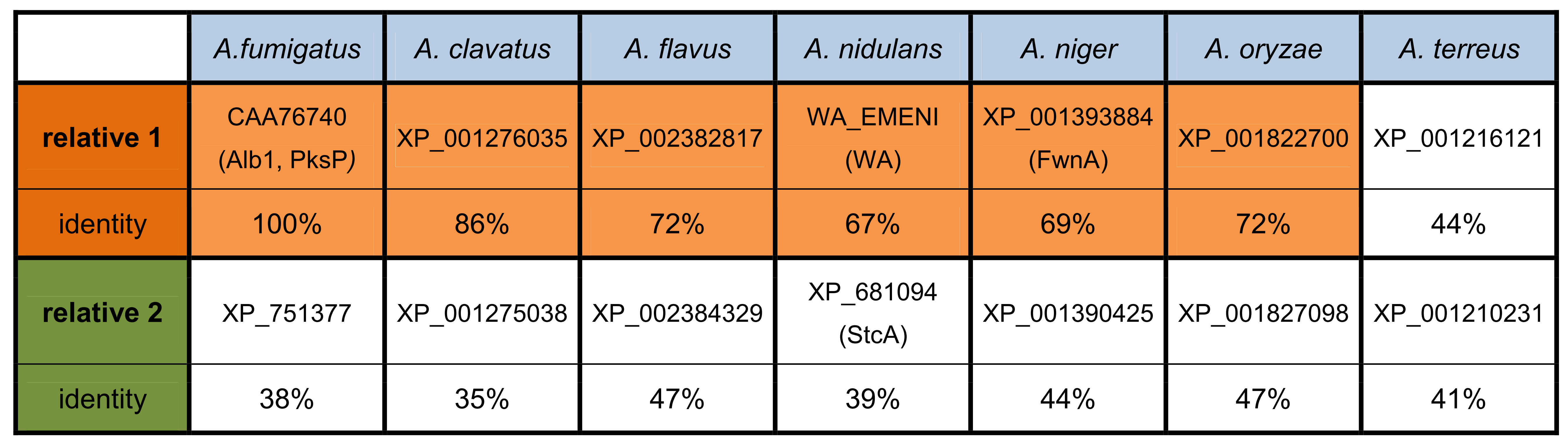


**Figure S8: Conservation of polyketide synthases responsible for conidia coloration among different *Aspergillus* species.** The polyketide synthase PksP from *A. fumigatus* was used as a template in BLASTP analyses against genomes from different Aspergilli. All identity values (in %) regard to PksP. With the exception of *A. terreus* all other Aspergilli contain a highly homologous polyketide synthase (67 to 86% identity). The second most closely related polyketide synthase present in genomes of Aspergilli (bottom lane) shows between 35 and 47% identity to PksP, but seems, at least for *A. fumigatus,* *A. nidulans*, and *A. niger*, not involved in conidia coloration. *A. terreus* contains polyketide synthases with high identity to these second polyketide synthases, but not to PksP. Deletion of the two polyketide synthases presented for *A. terreus* did not alter the color of *A. terreus* conidia (data not shown).
