# Supplementary material for: Persistence versus Escape: Aspergillus terreus and Aspergillus fumigatus Employ Different Strategies during Interactions with Macrophages
Source: PLoS One. 2012 Feb 3;7(2):e31223. doi: 10.1371/journal.pone.0031223 (PMC3272006; doi:10.1371/journal.pone.0031223)
Supplement: Figure S9 — Determination of naphthopyrone from A. terreus wA . (DOC) [file pone.0031223.s009.doc]

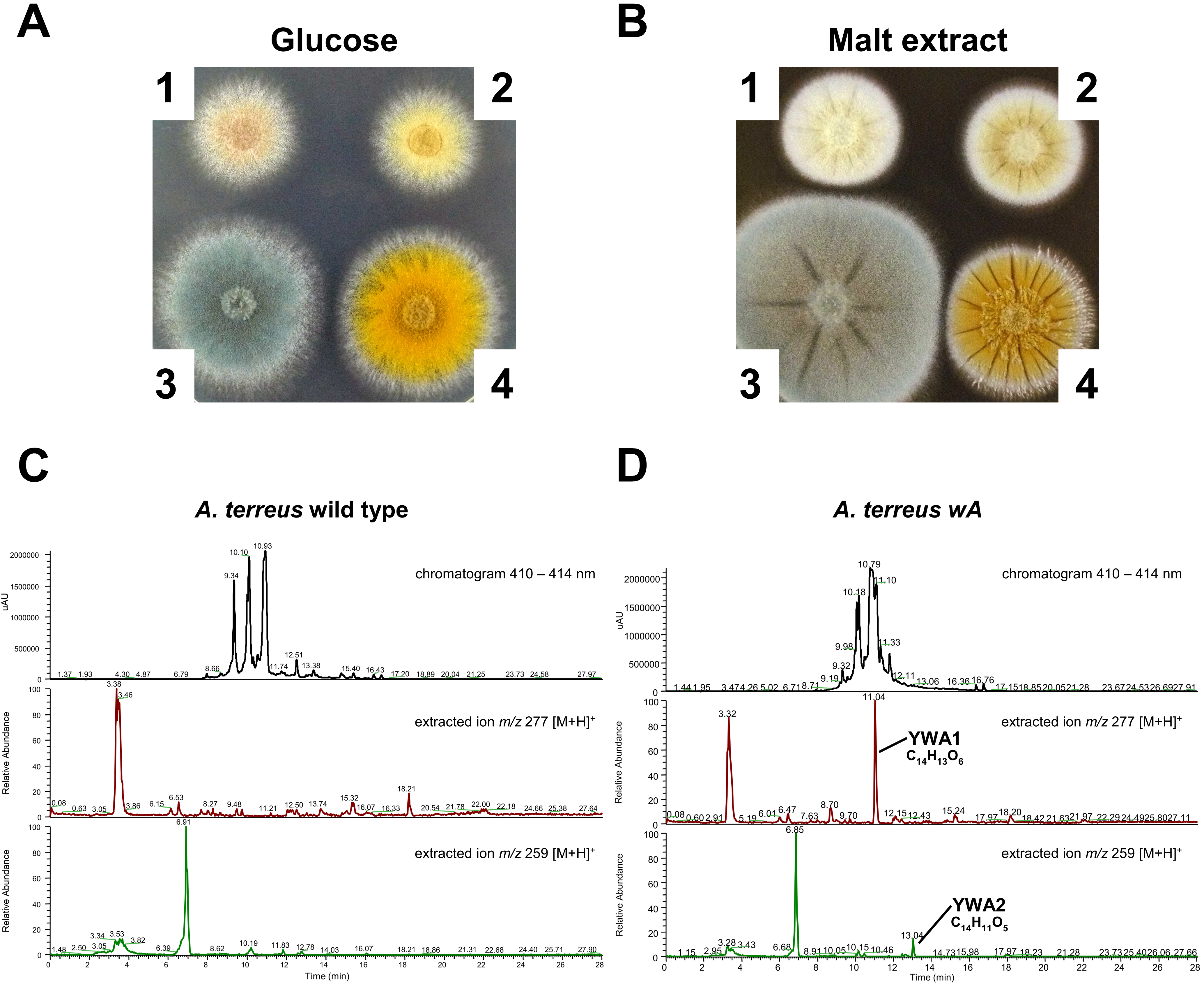


**Figure S9: Determination of naphthopyrone from *A. terreus wA*.** (A) and (B) show the colony appearance of *A. terreus* wild type (1), *A. terreus wA* (2), *A. fumigatus* wild type (3), and *A. nidulans* yA (4) on solid glucose minimal medium (Glucose) and malt extract agar (Malt extract), respectively. The colony appearance of the *A. terreus wA* strain shifts to yellow. (C) and (D): Chromatogram at 410 – 414 nm (upper lane) showing secondary metabolites extracted from conidia of *A. terreus* wild type (C) and *A. terreus* *wA* (D). HR-ESI mass spectrometry was used to detect the masses of two naphthopyrone derivatives as identified from *A. nidulans*. In the middle lane the extracted ion chromatogram *m/z* 277 [M+H]^+^ is shown, which is characteristic for the naphthopyrone derivative YWA1. The bottom lane shows an ion extraction chromatogram at *m/z* 259 [M+H]^+^, which is characteristic for a dehydrated form of naphthopyrone denoted YWA2. The peak denoted YWA1 at a retention time of 11.04 min revealed a mass of 277.0707 amu and was calculated by the software Thermo Xcalibur 2.1 to possess the molecular formula C_14_H_13_O_6_ (error of 2.15 ppm). The peak denoted YWA2 at a retention of 13.04 min showed a mass of 259.0601 amu with a calculated molecular formula of C_14_H_11_O_5_ (error 1.97 ppm). This confirms the successful production of naphthopyrone in the *wA* expressing strain.
