# Supplementary material for: Persistence versus Escape: Aspergillus terreus and Aspergillus fumigatus Employ Different Strategies during Interactions with Macrophages
Source: PLoS One. 2012 Feb 3;7(2):e31223. doi: 10.1371/journal.pone.0031223 (PMC3272006; doi:10.1371/journal.pone.0031223)
Supplement: Figure S10 — Acidification of phagolysosomes in primary macrophages containing A. terreus wA . (DOC) [file pone.0031223.s010.doc]

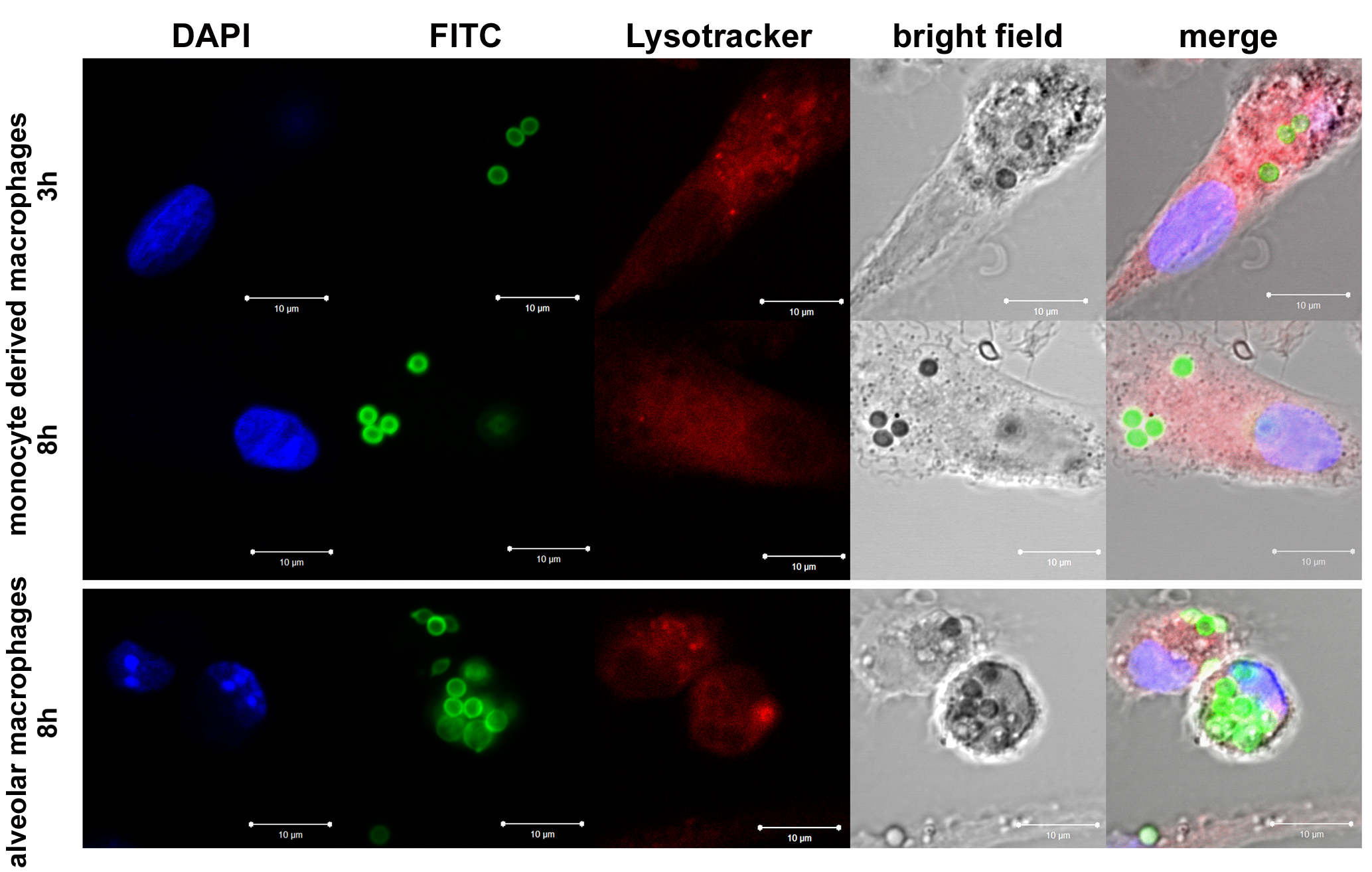


**Figure S10. Acidification of phagolysosomes in primary macrophages containing *A. terreus wA*.** FITC labeled conidia were used. Yellow signal in the merged pictures indicate co-localization of conidia with the lysotracker stain. All lanes show representative fluorescence microscopy pictures. Blue: DAPI (nucleus); green: FITC labeled conidia; red: Lysotracker. Bars represent a size of 10 µm. The strains are indicated on the left side of the lanes.
