# Supplementary material for: Persistence versus Escape: Aspergillus terreus and Aspergillus fumigatus Employ Different Strategies during Interactions with Macrophages
Source: PLoS One. 2012 Feb 3;7(2):e31223. doi: 10.1371/journal.pone.0031223 (PMC3272006; doi:10.1371/journal.pone.0031223)
Supplement: Figure S11 — Histology and quantification of inflammation in mice infected with A. terreus wild type or A. terreus wA . (DOC) [file pone.0031223.s011.doc]

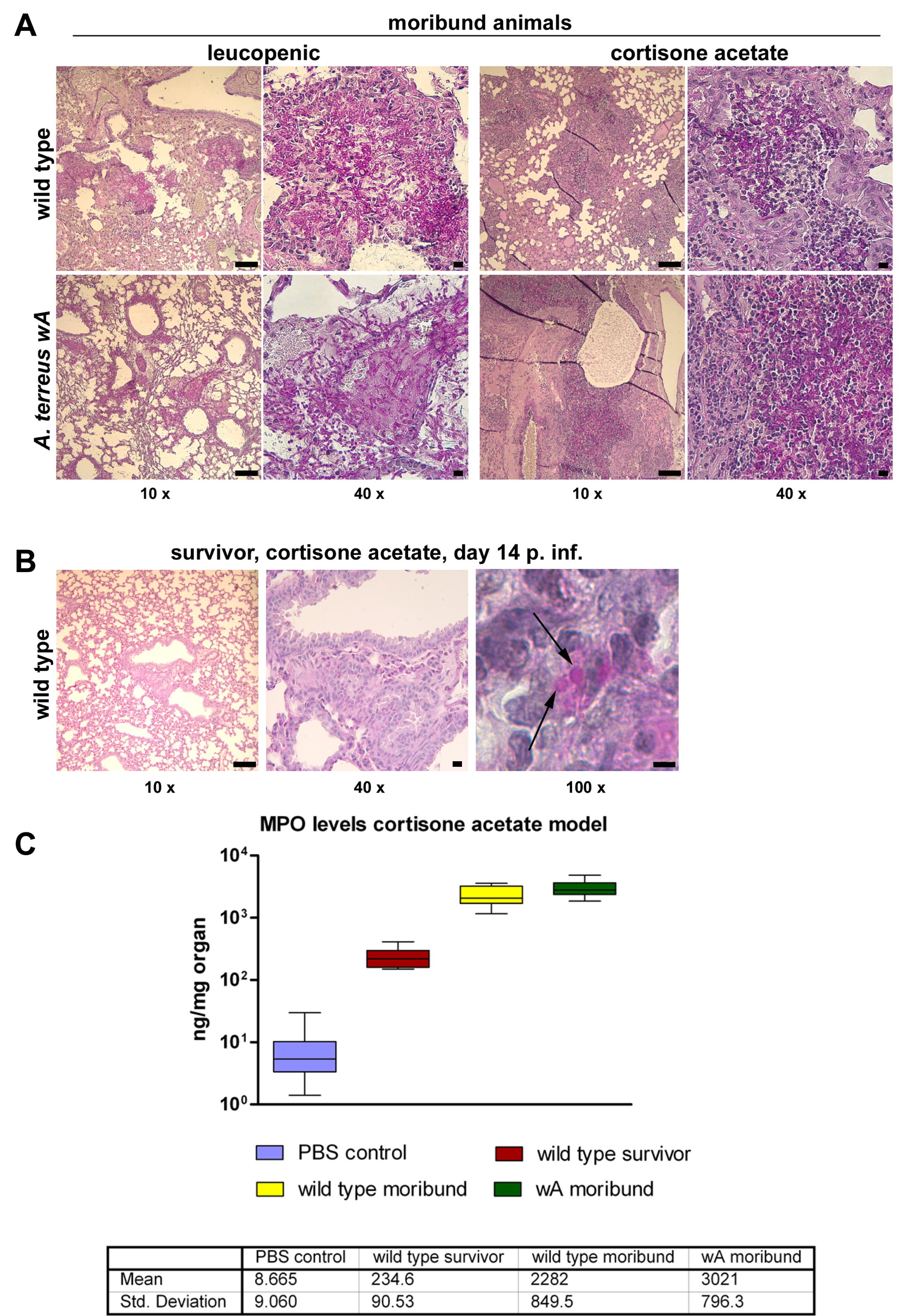


**Figure S11. Histology and quantification of inflammation in mice infected with *A. terreus* wild type or *A. terreus wA*.** (A) Lung histology of moribund mice either rendered leucopenic or treated with cortisone acetate and infected with *A. terreus* and *A. terreus wA*, respectively. In moribund leucopenic mice both strains display massive tissue invasion without recruitment of immune cells. In moribund mice treated with cortisone acetate both fungal strains formed tissue-invading hyphae, but tissue invasion and immune cell recruitment appears more severe with the *A. terreus wA* strain; scale bars represent 100µm (10×) and 10µm (40×). (B) Subsequent magnifications of a lung section from a corticosteroid treated mouse surviving infection with the *A. terreus* wild-type strain. No fungal mycelium is visible, but ungerminated conidia are present within macrophages, scale bars represent 100µm (10×) and 10µm (40×; 100×). (C) Levels of myeloperoxidase (MPO) in corticosteroid treated mice. MPO levels were determined from mock infected control mice, mice infected with *A. terreus* wild type that either succumbed to infection, or survived, and mice infected with *A. terreus wA* that succumbed to infection. Mean values and standard deviations are shown in the underlying table. In moribund animals and regardless the infecting strain MPO levels are at least 250 times higher than in mock infected animals. In surviving animals MPO levels are 10 times lower than in moribund animals pointing to a resolution of inflammation.
